# Graded calls of the smallest terrestrial mammal, the Etruscan shrew, living in a closed habitat

**DOI:** 10.1016/j.isci.2024.111297

**Published:** 2024-11-01

**Authors:** Alexandra Langehennig-Peristenidou, Felix Felmy, Marina Scheumann

**Affiliations:** 1 Institute of Zoology, University of Veterinary Medicine Hannover, Hannover, Germany

**Keywords:** bioacoustics, evolutionary biology, zoology

## Abstract

Graded call types predominate in species inhabiting open habitats with complex social systems, whereas discrete call types predominate in species with simple social systems living in closed habitats. This study aims to establish the vocal repertoire of Etruscan shrews, the smallest terrestrial mammal, which lives in pairs in closed habitats. Through various behavioral experiments, vocalizations were recorded and analyzed using unsupervised soft clustering, identifying four call types, one of which exhibited gradation. These calls were present in both pups and adults, showing age-related acoustic differences. One call type was observed during socio-positive behavior, with higher call rates during female-male interactions, while the others occurred during socio-negative contexts, with higher call rates for animals housed in pairs. Delivering the first detailed insight into Etruscan shrew vocal behavior, we demonstrated that the smallest terrestrial mammal possesses graded and discrete call types, regardless of its social system and habitat.

## Highlights

• Graded calls are produced by species in open habitats with complex social systems
• Etruscan shrews emitted discrete and graded calls while living in pairs in closed habitats
• Pups and adults produced the same call types, albeit with age-related differences
• Different calls were produced during socio-positive and socio-negative contexts

## Graphical abstract

**Figure:**
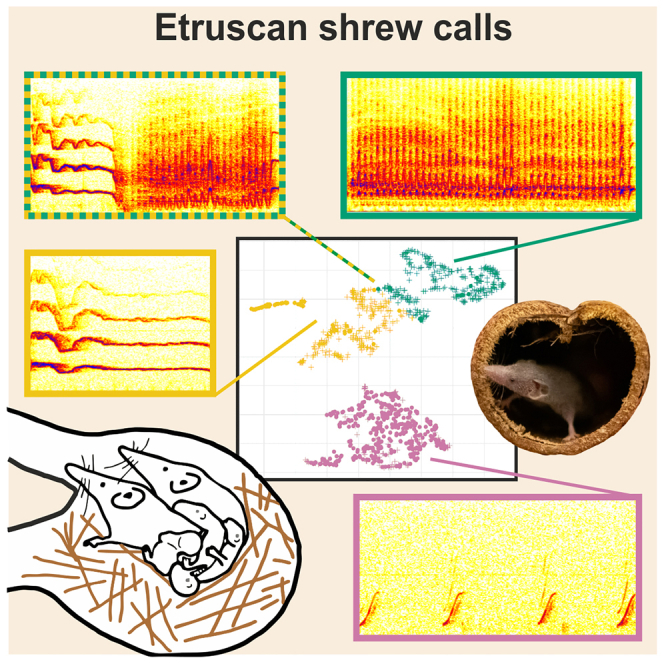

## Introduction

By uttering different call types, animals can signal the different contexts the sender is exposed to.^1^ Calls may be produced in response to external stimuli, which encompass alarm and food calls, or during social interactions with conspecifics.^2^ Over long distances, vocalizations can be used for intergroup spacing,^3^ to maintain contact between conspecifics,^4^ or to attract distant mating partners.^5^ In close-range, vocalizations can also be emitted to coordinate affiliative, aggressive or mating interactions.^2^ Thereby, calls can contain information regarding the physical characteristics, the emotional state and the social relationships of the sender.^6^^,^^7^^,^^8^^,^^9^ The production of diverse call types, each serving distinct functions, highlights the importance of studying vocal repertoires in a context-dependent manner to better grasp the complexity of animal vocal communication.^10^

Vocal repertoires catalog the various call types utilized by a species through descriptions of their acoustic structure and contextualized by reporting the context in which they are produced.^10^ By containing both adult and offspring vocalizations, vocal repertoires can provide a first insight into the vocal development of the respective species. During infancy, offspring often produce specific call types, primarily to attract the attention of the caregiver.^11^ These infant specific calls may diminish or disappear as the offspring develop e.g., pup isolation calls decrease with pup age (e.g.,^6^^,^^10^^,^^12^^,^^13^^,^^14^^,^^15^), or can gradually develop to adult-specific call types, solely produced by adults (e.g.,^16^^,^^17^^,^^18^). This development can occur either due to vocal learning or as a result of physical maturation.^9^^,^^19^^,^^20^^,^^21^ Furthermore, due to the gradual development of the vocal production apparatus, pups may fail to produce well-formed, mature adult calls, instead exhibiting a high variability in their vocalisations.^22^^,^^23^ For call types produced by vibrations of the vocal folds that are shared between adults and pups, notable differences in their acoustic parameters have been reported: the fundamental frequency (F0) is typically higher in pup calls compared to adult calls,^24^^,^^25^^,^^26^ while adult vocalizations are usually longer.^21^^,^^27^^,^^28^ These differences are a consequence of the gradual increase in the size of their vocal production system during ontogeny.^21^^,^^26^

Vocal repertoires can be graded, discrete, or a mixture of both depending on the acoustic variations among call types.^29^^,^^30^ Discrete call types show a distinct acoustic structure with no intermediates between each other.^31^ Graded call types lack clear boundaries between them and are characterized by high acoustic variation.^32^ Thereby, it is possible that the different call types of a species show varying degrees of gradation based on their contextual usage, with e.g., contact and threat calls being more variable than long-distance or alarm calls.^31^ Regarding the evolution of the various types of vocal repertoires, it has been hypothesized that the degree of variation in a vocal repertoire of a species is determined by its predation pressure, habitat and sociality.^31^^,^^33^ Discrete call types are typically observed when additional cues are not feasible, such as in closed habitats or during long distance calls,^31^^,^^33^ and/or in species with simple social structures.^30^^,^^31^ In addition, species with predator-specific defense strategies tend to have discrete alarm calls to minimize ambiguity for recipient conspecifics.^34^ Graded call types predominate in animal species inhabiting open areas, where communication occurs frequently and at a close range with conspecifics.^31^^,^^33^ Thus, they can accompany their calls with visual and contextual signs. Concerning the effect of sociality on vocal repertoires, in general, the number of cell types present in a vocal repertoire has been suggested to reflect the complexity of their social organization, with species exhibiting a more intricate social organization producing a higher number of distinct call types.^35^^,^^36^^,^^37^^,^^38^^,^^39^^,^^40^ The same is also the case with gradation, with graded calls allowing the encoding of additional information, e.g., the motivational state or the arousal level of the sender.^41^ Thus, gradation is more often present in social complex living species and especially in call types with an important social function.^33^

In this study, we aim to explore the vocal repertoire of the Etruscan shrew (*Suncus etruscus*), a pair-living species, which lives underground in burrows or under stones.^42^^,^^43^ Thus, both the structure of their habitat, which does not allow the use of visual and temporally precise contextual signs, as well as their simple social structure suggest that they would possess a discrete vocal repertoire with a limited number of call types. Etruscan shrews are suggested to represent an ancestral mammalian model for hearing^44^^,^^45^ due to their small body and brain size,^46^^,^^47^ along with the basal structure of the middle ear and a low number of hair cells in related species.^48^^,^^49^ Thus, it is an especially valuable animal model for studying the co-evolution of hearing and vocal communication.

Etruscan shrews (*Suncus etruscus*) are the smallest extant terrestrial mammal, weighing on average 2–3 g.^43^^,^^50^ They belong to the family Soricidae (subfamily: Crocidurinae) and the order Eulipotyphla.^42^^,^^51^^,^^52^ Due to their small size and short fur, they have a high metabolism and possess an imperfect thermoregulation, being very sensitive to variations of the surrounding temperatures.^53^ In addition, due to their high energetic demands, Etruscan shrews exhibit polyphasic patterns, with alternating periods of activity and rest throughout the day.^42^ They typically live in family groups (breeding pair with their unweaned pups) and display social monogamy,^42^ while in captivity they are kept in same-sex groups or breeding pairs.^43^ Known for their aggressiveness toward unfamiliar individuals, Etruscan shrews either avoid or engage in violent confrontations with them.^42^^,^^53^ However, intraspecific intolerance is not absolute; cohabitation between unrelated individuals of the same sex has been observed, while pairs formed during the breeding season stay together for the rest of their lives without being aggressive toward each other. A typical behavior of Crocidurinae also practiced by *S. etruscus* is caravanning.^42^^,^^54^ Caravanning involves pups immediately attaching to the mother’s fur when the nest is disturbed, forming a caravan by biting onto each other’s fur. This behavior, which starts around day 9 or 10 after birth and coincides with the opening of the acoustic meatus, seems to be an innate response for protection and escape.^42^ Despite their significance as a model for hearing research,^44^^,^^45^ very little is known about the vocal communication of Etruscan shrews. Spitzenberger^53^ mentions four potential call types uttered during pup isolation, mating, aggression, and defense, but does not deliver a detailed description. Sonograms are available for only two call types from studies by Hutterer and colleagues: a call with a tremolo-structure and a mean frequency of 16.4 kHz considered a defensive call^55^ and a long-duration call with an almost constant frequency of around 17 kHz produced during male-female interactions.^56^

The goal of this study is to get an insight into the vocal repertoire of the Etruscan shrews by analyzing audio and video recordings of social confrontation and pup vocalization experiments. The calls are to be classified based on their acoustic properties using multivariate statistical methods, while the context during which they are emitted is to be reported. Thereby, we want to investigate whether Etruscan shrews possess a discrete vocal repertoire, as suggested by their ecology and social structure. In addition, we aim to identify the acoustic parameters of the vocalizations which differ between adults and pups. Finally, we will explore whether differences in housing types, familiarity, or the sex composition of confronting dyads influence the number of calls produced.

## Results

### UMAP and unsupervised cluster analysis

**Figure 1:**
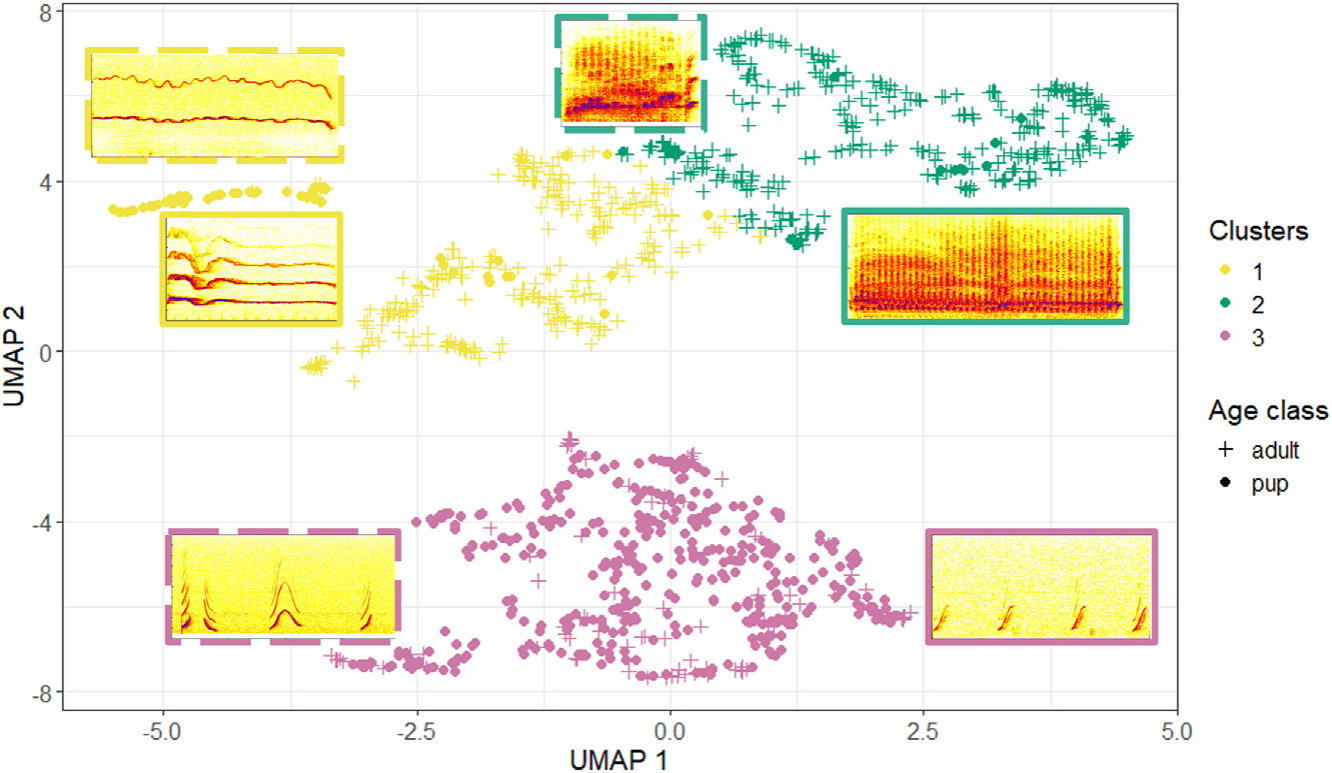
Bi-dimensional representation of the acoustic data (UMAP) Clusters identified by the FCM are shown in different colors, while the age groups are indicated in different symbols. Examples of sonograms from each cluster are shown in the same color as their corresponding cluster. Sonograms with solid lines represent adult calls and those with dotted lines represent pup calls.

We conducted social confrontation and pup vocalizations experiments with 45 Etruscan shrews, which were audio and videotaped. We measured 22 acoustic parameters to describe the tempo-spectral structure of 1131 high-quality calls (679 adult calls, 452 pup calls; Table 1). To ensure the subjective classification of the calls into call types, we performed an unsupervised cluster analysis. Thereby, we utilized UMAP (Uniform Manifold Approximation and Projection), a dimensionality reduction technique, as a pre-processing step. Since the visual inspection of the calls implied a gradation between them, we applied a soft clustering algorithm, the Fuzzy c-means (FCM). The UMAP projection using the 1131 high-quality calls revealed two distinct clouds, as shown in Figure 1: a compact cloud located at negative UMAP2 scores and a larger, more dispersed cloud at positive UMAP2 scores, displaying a gradient of data points. This gradient suggests a continuous variation or a spectrum within this cloud. Figure 1 illustrates the results of the FCM along with the UMAP analysis, with the FCM configured to identify three clusters within the data. Cluster 1 and Cluster 2 coincided with the larger cloud indicated by the UMAP, while Cluster 3 corresponded with the more compact cloud at the bottom of the UMAP space. Cluster 1 included mainly long tonal calls (duration: 0.16 ± 0.07 s, voiced percentage, i.e., percentage of voiced frames of a call: 81 ± 23%; N_calls_ = 323), whereas Cluster 2 consisted of mainly non-tonal calls (voiced percentage: 15 ± 18%; N_calls_ = 343). Thereby, Clusters 1 and 2 indicated a gradation, each containing calls with both tonal and non-tonal parts. Cluster 3 consisted of tonal calls (voiced percentage: 97 ± 7%; N_calls_ = 465), which were on average shorter than those of Cluster 1 (duration: 0.03 ± 0.02 s). All clusters were emitted by both adults and pups. Thereby, more than 80% of the calls from Clusters 1 and 2 were obtained from adults, whereas 81% of the calls from Cluster 3 were from pups. The mean and the standard deviation of selected acoustic parameters of the clusters can be seen in Table S1 in the supplementary.

**Table 1:** Description of measured acoustic parameters

Parameter	Definition	Availability
**Time-related parameters**		

Duration [s]	Time between the onset and the offset of a call.	All calls
Time of peak amplitude [s]	Time between the onset of a call and the peak amplitude.	All calls
Time of minimum fundamental frequency [s]	Time between the onset and the minimum of the fundamental frequency.	Tonal
Time of maximum fundamental frequency [s]	Time between the onset and the maximum of the fundamental frequency.	Tonal

**Tonality-related parameters**		

Voiced percentage [%]	Percentage of voiced frames of a call; it is calculated as the ratio of voiced frames and the sum of all frames of a call and is semi-automatically defined. A frame was defined as voiced if the fundamental frequency was in the corresponding frame clearly visible.	All calls
Harmonics-to-noise ratio [dB]	Represents the overall periodicity of the voice signal by quantifying the ratio between the periodic (harmonic part) and aperiodic (noise) components.	All calls
Wiener entropy [dB]	Ratio of geometric to arithmetic energy.	All calls

**Source-related spectral parameters**		

Minimum fundamental frequency [kHz]	Minimum fundamental frequency of a call.	Tonal
Maximum fundamental frequency [kHz]	Maximum fundamental frequency of a call.	Tonal
Mean fundamental frequency [kHz]	Mean fundamental frequency of a call.	Tonal
Standard deviation of the fundamental frequency [kHz]	Standard deviation of the fundamental frequency of a call.	Tonal
Mean slope [kHz/s]	Mean absolute slope of the fundamental frequency; it refers to the sum of the absolute values of the slopes between the different detection points of the pitch divided by time.	Tonal

**Filter-related spectral parameters**		

Center of gravity [kHz]	Mean frequency of the spectrum of a call.	All calls
Standard deviation [kHz]	Measure for how much the frequencies in a spectrum deviate from the center of gravity.	All calls
Skewness	Difference between the spectral distribution below and above the center of gravity.	All calls
Kurtosis	Measure of how much the shape of a spectrum around the center of gravity is different from a Gaussian shape.	All calls

**Formant-like resonance parameters**		

Frequency of first formant [kHz]	Mean frequency of the first formant of a non-tonal call.	Non-tonal
Bandwidth of first formant [kHz]	Difference between the upper and lower frequency of the first formant of a non-tonal call.	Non-tonal
Frequency of second formant [kHz]	Mean frequency of the second formant of a non-tonal call.	Non-tonal
Bandwidth of second formant [kHz]	Difference between the upper and lower frequency of the second formant of a non-tonal call.	Non-tonal
Frequency of third formant [kHz]	Mean frequency of the third formant of a non-tonal call.	Non-tonal
Bandwidth of third formant [kHz]	Difference between the upper and lower frequency of the third formant of a non-tonal call.	Non-tonal

Availability describes for which calls the corresponding acoustic parameter was available.

**Figure 2:**
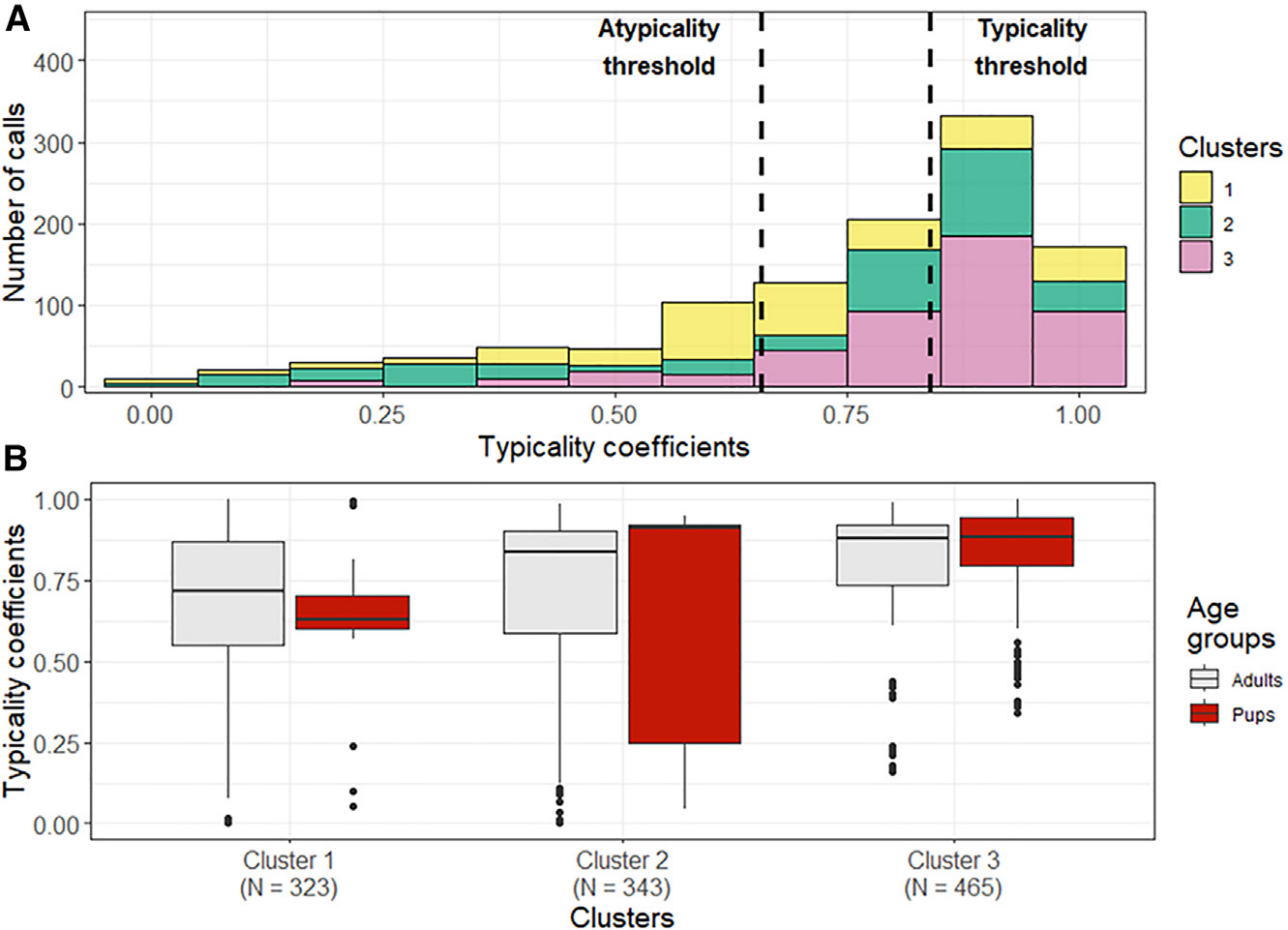
Distribution of the typicality coefficients acquired by the FCM (A) Histograms illustrating the frequency of the typicality coefficients for the acquired clusters. Dotted lines depict the typicality and atypicality thresholds, defined based on the methodology. (B) Boxplots illustrates the typicality coefficients of each cluster for the age groups of adults and pups. The boxplots represent lower and upper quartiles; the thick black line is the median and the whiskers are the non-outlier range; N: number of calls per cluster.

To investigate whether the FCM supports the observation that gradation is present in the calls of Etruscan shrews, we calculated typicality coefficients and established typicality and atypicality thresholds (confer to STAR Methods for precise calculation). Based on the atypicality and typicality thresholds, the calls were categorized into three typicality categories: calls with typicality coefficients higher than the typicality threshold are considered typical, calls with typicality coefficients lower than the atypicality threshold are considered atypical, while calls with typicality coefficients lower than the typicality threshold and higher than the atypicality threshold are neither typical or atypical (from now on referred as neither). The histogram illustrating the distribution of the typicality coefficients (Figure 2A; histograms for the clusters separately can be found in Figure S1 in the supplementary) revealed that nearly half of the calls were classified as typical, with Cluster 3 being the most “typical” cluster (62%; Table S2, Figure S2 in the supplementary). However, most of the calls of Cluster 1 (45%) and many of the calls of Cluster 2 (30%) were classified as atypical. For Cluster 1, 40% of the adult and 65% of the pup calls were atypical calls. Atypical pup calls often had gaps in their fundamental frequency. For Cluster 2, 49% of the adult and 67% of the pup calls were classified as typical, whereas over 30% were classified as atypical, containing both tonal and non-tonal elements. To test whether differences in the typicality of the calls may be a consequence of age-related differences, LMEs were calculated testing the effect of age group (adults, pups) on the typicality coefficients for each cluster. For all three clusters, no significant effect of age group on the typicality coefficients was found (t ≤ |1.80|, *p* ≥ 0.085; Figure 2B, Table S3). Thus, differences in the typicality cannot be explained by age group.

Visual inspection of the calls from Clusters 1 and 2 indicated that atypical calls exhibited a graded structure consisting of both tonal and non-tonal parts. To quantitatively assess our visual observation, we conducted linear mixed-effects models (LMEs) to examine the differences in voiced percentage across typicality categories (typical, atypical, neither). The results revealed a significant effect of typicality on voiced percentage for both Cluster 1 and Cluster 2 (LMEs: χ^2^ > 247.18, df = 2, *p* < 0.001), indicating that the voiced percentage significantly varies among the typicality categories (typical, atypical, neither). Pairwise comparisons using the least-squares means method (lsmeans) revealed that typical calls had a significantly higher voiced percentage in Cluster 1 and a significantly lower voiced percentage in Cluster 2 compared to atypical calls (lsmeans: t ≥ |15.01|, *p* < 0.001; Figure S3).

**Figure 3:**
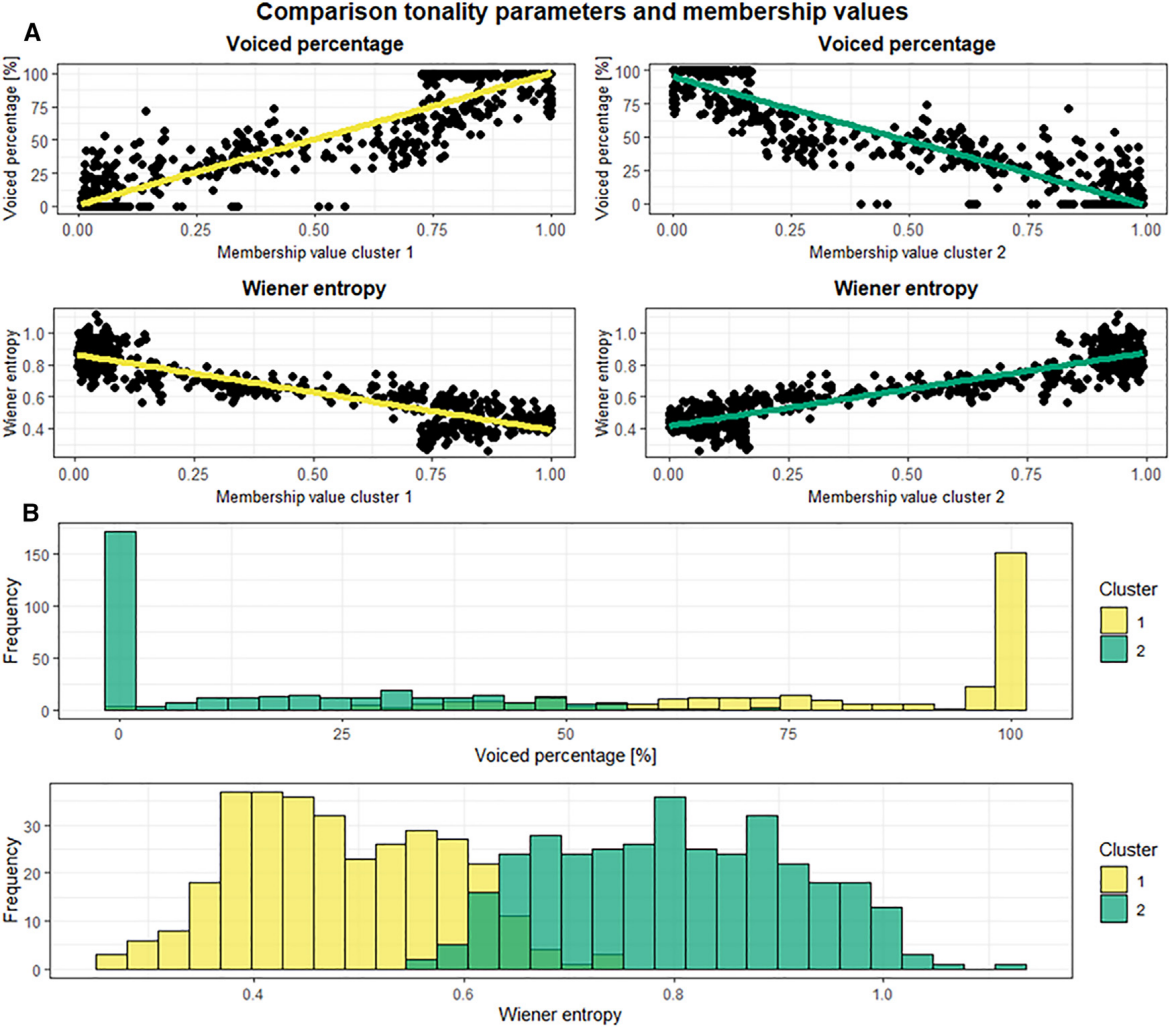
Comparison of tonality parameters with the membership values and the closest hard clusters acquired by the FCM for Cluster 1 and Cluster 2 (A) Scatterplots highlighting the relationship between the tonality-related acoustic parameters (voiced percentage, wiener entropy) with the membership values for Cluster 1 and Cluster 2. (B) Histograms of the distribution of the tonality-related acoustic parameters (voiced percentage and wiener entropy) for calls clustered into Cluster 1 and Cluster 2.

The classification of calls containing tonal and non-tonal parts of Clusters 1 and Cluster 2 as atypical emphasizes the graded nature of some of the calls of these two clusters. To test whether this gradation may be a manifestation of differences in the tonality of the calls, LMEs were calculated testing the effect of tonality-related acoustic parameters (voiced percentage, wiener entropy) on the membership values of calls of Cluster 1 and Cluster 2. Calls with higher tonality had significantly higher values for Cluster 1 (membership value cluster 1/voiced percentage: t = 52.78, df = 636, *p* < 0.001; membership value cluster 1/wiener entropy: t = −42.38, df = 636, *p* < 0.001; Figure 3A, Table S4), whereas calls with lower tonality values had significantly higher membership values for Cluster 2 (membership value cluster 2/voiced percentage: t = −56.42, df = 636, *p* < 0.001; membership value cluster 2/wiener entropy: t = 45.70, df = 636, *p* < 0.001; Figure 3A, Table S4). The histograms of the distributions of the tonality-related acoustic parameters grouped by cluster showed an overlap between Cluster 1 and Cluster 2 for voiced percentage and wiener entropy (Figure 3B), indicating that a definite boundary for separating graded calls between Cluster 1 and Cluster 2 could not be established.

### Definition of call types

**Figure 4:**
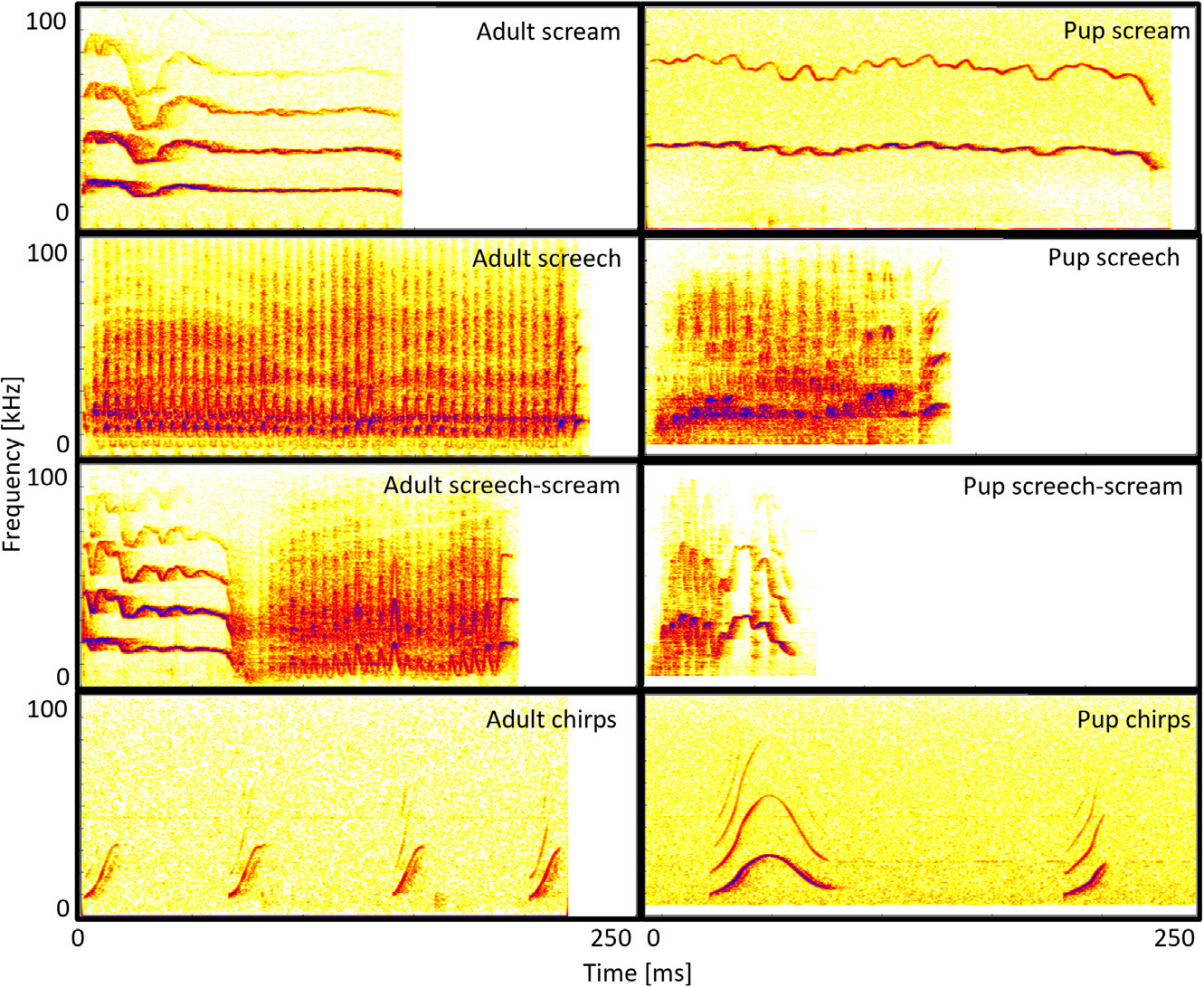
Sonograms of screams, screeches, chirps, and screech-screams described for the Etruscan shrews Examples of adult and pup calls are provided.

Based on the results of the FCM, three clusters and a gradation between Clusters 1 and 2 were observed. Graded calls were predominantly atypical calls from Cluster 1 and Cluster 2. Thus, calls from Cluster 1 with a voiced percentage below 95% and calls from Cluster 2 with a voiced percentage above 0% were treated as a separate call type. Based on the nomenclature used for other shrew species (e.g., Asian house shrew (*Suncus murinus*): Schneiderová,^57^ piebald shrew (*Diplomesodon pulchellum*): Volodin et al.^26^), four different call types were distinguished: pure tonal calls from Cluster 1 were termed as screams, pure noisy calls from Cluster 2 were termed screeches, the graded calls of Clusters 1 and 2, which combined both tonal and noisy elements, were termed screech-screams and calls from Cluster 3 were termed chirps. Exemplary spectrograms of the call types can be found in Figure 4 while the mean and the standard deviation of selected acoustic parameters of the call types can be seen in Table S5 in the supplementary. LMEs comparing the acoustic parameters available for all calls among the described call types showed significant differences in all acoustic parameters (χ^2^ ≥ 21.28, df = 3, *p* < 0.001; Table S6).

**Screams** were long solely tonal calls (duration: 0.16 ± 0.07 s; voiced percentage: 99 ± 1%; N_calls_ = 173) which showed only a weakly modulated contour of the fundamental frequency (mean fundamental frequency: 24.49 ± 5.92 kHz; standard deviation of the fundamental frequency: 2.47 ± 0.92 kHz). They were significantly longer than Chirps and Screeches (lsmeans: t ≥ |3.53|, *p* ≤ 0.002; Table S7) and showed the highest values for the center of gravity compared to the other call types (lsmeans: t ≥ |3.44|, *p* ≤ 0.003; Table S7), while they had a significantly higher voiced percentage than Screeches and Screech – screams (lsmeans: t ≥ |44.34|, *p* < 0.001; Table S7). **Screeches** were characterized by their tremolo structure and smeared fundamental frequency, with no tonal elements (voiced percentage: 0 ± 0%; N_calls_ = 171). Screeches (duration: 0.11 ± 0.08 s) were significantly longer than Chirps but shorter than Screams and Screech – screams (lsmeans: t ≥ |3.53|, *p* ≤ 0.002; Table S7). The mean spectral frequency (center of gravity: 22.94 ± 3.63 kHz) was significantly higher compared to Chirps but lower than for Screams (lsmeans: t ≥ |4.44|, *p* < 0.001; Table S7). Thereby, Screeches showed a significantly lower voiced percentage than Chirps, Screams, and Screech – screams (lsmeans: t ≥ |31.69|, *p* < 0.001; Table S7). **Screech – screams** were vocalizations combining elements resembling the screams and elements that resemble the screeches with usually a long duration (duration: 0.15 ± 0.06 s) and a mean spectral frequency comparable to the one of the screeches (center of gravity: 24.44 ± 2.20 kHz). The duration of Screech – screams was significantly longer than for Chirps and Screeches (lsmeans: t ≥ |5.86|, *p* < 0.001; Table S7) and they had a lower tonality than Chirps and Screams (lsmeans: t ≥ |42.26|, *p* < 0.001; Table S7). The center of gravity of Screech – screams was significantly higher than for Chirps but lower than for Screams (lsmeans: t ≥ |3.44|, *p* ≤ 0.003; Table S7). **Chirps** were short tonal calls (duration: 0.03 ± 0.02 s; voiced percentage: 97.35 ± 6.89%; N_calls_ = 465), with a lower mean fundamental frequency than screams which were more modulated (mean fundamental frequency: 19.87 ± 4.41 kHz; standard deviation of the fundamental frequency: 5.54 ± 2.31 kHz). Chirps were significantly shorter and had a significantly lower center of gravity than Screeches, Screams, and Screech – screams (lsmeans: t ≥ |6.81|, *p* < 0.001; Table S7) whereas they showed a higher voiced percentage than Screeches and Screech – screams (lsmeans: t ≥ 42.26, *p* < 0.001; Table S7).

### Comparisons of the acoustic parameters between age groups

**Figure 5:**
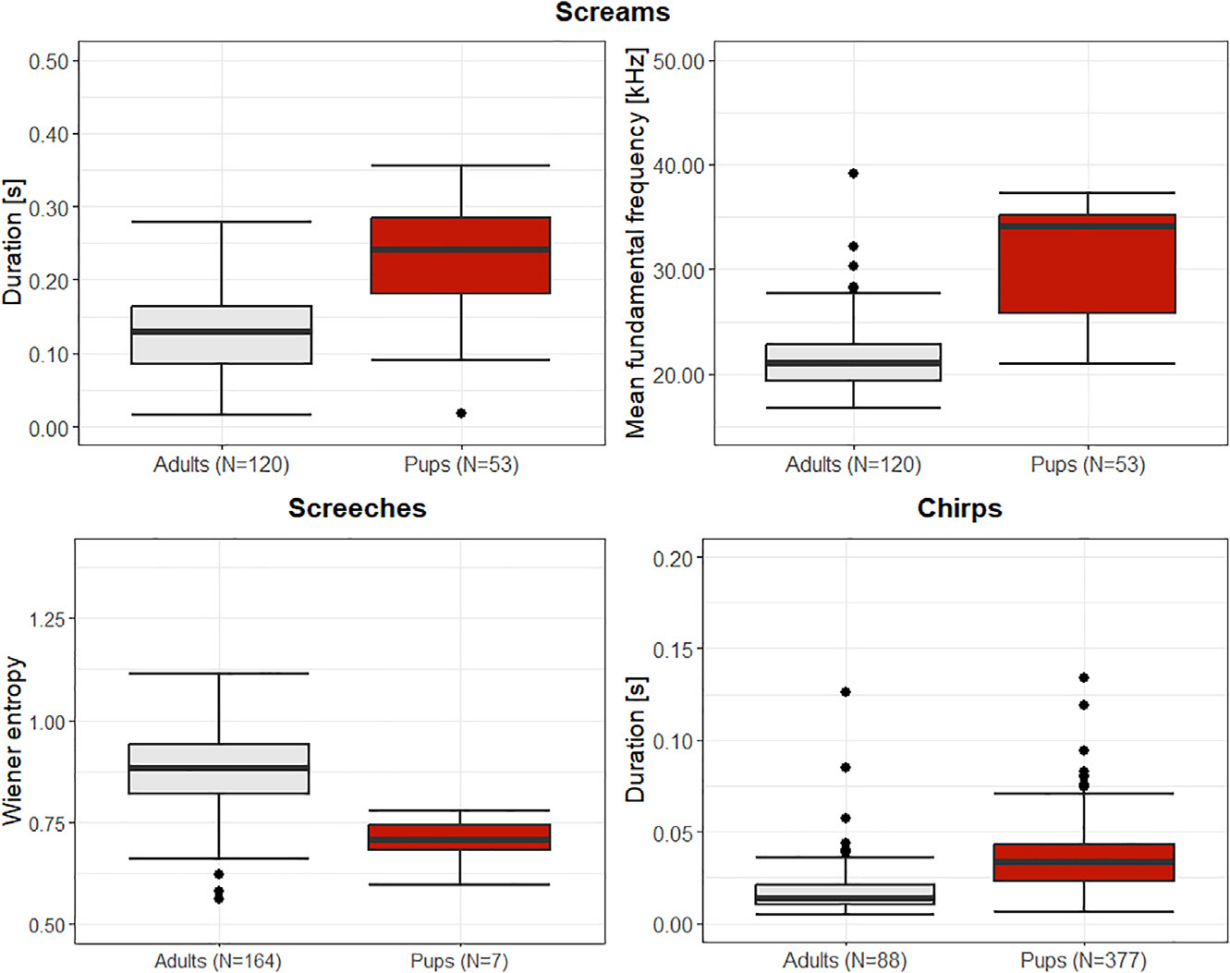
Boxplots comparing a selection of acoustic parameters between adults and pups for the clusters acquired by the FCM The boxplots represent lower and upper quartiles; the thick black line is the median and the whiskers are the non-outlier range. N: number of calls per age group.

To investigate whether age-dependent differences are present in the different call types described in this study, LMEs were calculated. We found that the screams had the most differences between adults and pups. There, 9 out of 16 acoustic parameters differed significantly between the two age groups (Fisher Omnibus test: χ^2^ = 127.52, df = 32, *p* < 0.001; N_calls(adults)_ = 120, N_calls(pups)_ = 53; Table S8). Pup screams were thereby longer than adult calls, taking longer to reach the maximum amplitude or the time of minimum fundamental frequency (LMEs: t ≥ |4.41|, *p* ≤ 0.003). Furthermore, pup screams had a higher maximum and mean fundamental frequency and a higher mean slope in their contour compared to the adults (LMEs: t ≥ 3.53, *p* ≤ 0.010). Pups also produced calls with a higher tonality than adults (LMEs: harmonics-to-noise ratio (hnr): t = 2.51, *p* = 0.041; wiener entropy: t = −3.40, *p* = 0.011). Screeches had only a few age-dependent differences in their acoustic parameters, with only 3 out of 14 acoustic parameters differing significantly between adults and pups (Fisher Omnibus test: χ^2^ = 59.48, df = 28, *p* < 0.001; N_calls(adults)_ = 164, N_calls(pups)_ = 7; Table S8). Thereby, adult screeches had a lower tonality (LMEs: hnr: t = 4.07, *p* < 0.001; wiener entropy: t = −4.38, *p* < 0.001) and the frequency was more right skewed compared to the pups (LME: t = 2.84, *p* = 0.010). For screech-screams only 5 out of 15 acoustic parameters differed significantly between adults and pups (Fisher Omnibus test: χ^2^ = 76.47, df = 30, *p* < 0.001; N_calls(adults)_ = 307, N_calls(pups)_ = 15; Table S8). Thereby, as for the screeches, calls of the adults had a lower tonality (LMEs: voiced percentage: t = 2.37, *p* = 0.029; hnr: t = 4.90, *p* < 0.001; wiener entropy: t = −3.23, *p* = 0.004), but the screech-screams of the pups had a higher mean frequency of a spectrum (center of gravity: t = 3.51, *p* = 0.002) and a higher kurtosis (t = 3.33, *p* = 0.004). Finally, for the chirps, 5 out of 16 acoustic parameters differed significantly between adults and pups (Fisher Omnibus test: χ^2^ = 91.99, df = 32, *p* < 0.001; N_calls(adults)_ = 88, N_calls(pups)_ = 377; Table S8). Pups emitted longer chirps, taking longer to reach the maximum amplitude and the time of maximum fundamental frequency (LMEs: t ≥ |3.20|, *p* ≤ 0.004). In addition, the pup chirps had a lower minimum fundamental frequency (LMEs: t = −2.62, *p* = 0.016) and a higher standard deviation of the fundamental frequency (LMEs: t = 3.34, *p* = 0.003). Examples of differing acoustic parameters between adults and pups can be seen in Figure 5, while the detailed results for the comparisons for each call type and the mean acoustic parameters can be seen in Tables S8 and S9 in the supplementary respectively.

### Behavioral context analysis

For Etruscan shrew pups, the measured screams, screeches, and screech-screams were almost exclusively recorded during handling by the experimenter, whereas pup chirps were recorded when the pups were separated from their parents across all experimental conditions. For the adults, the situation was more complex, necessitating a detailed behavioral analysis of the adult confrontation experiments.

While only data from high-quality calls were used for the FCM and the comparison of the acoustic parameters, to account for all recorded vocalizations and thus provide a more accurate report of the functions of the different call types, all calls were visually classified based on the described vocal repertoire (Figure 4). To ensure that the visual classification of the calls matched the statistical classification described above, a Cohen’s kappa test was performed, which indicated an almost perfect agreement (κ = 0.964) between both classifications. As hardly any calls were produced during the closed-door phase (N_calls_ = 32), behaviors were solely coded for the open-door phase (N_calls_ = 2130).

**Figure 6:**
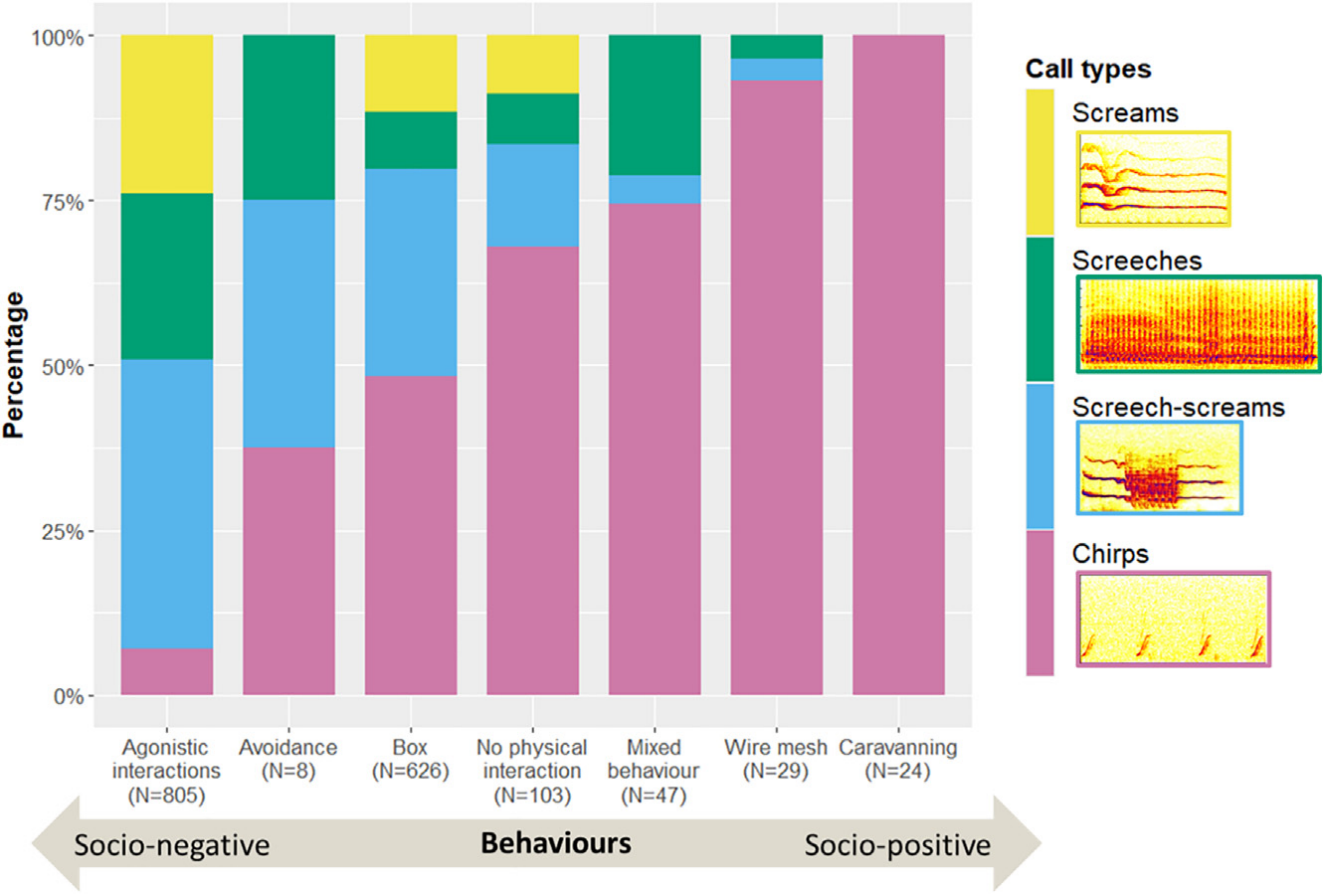
Shares of the call types for each coded behavior On the x axis the different coded behaviors are illustrated, while the different colors in the stacked bars demonstrate the shares of the call types in the sum of calls produced during the corresponding behavior. N: number of calls produced during the corresponding behavior.

In total, more than 75% of the vocalizations were produced when the animals were in proximity (Figure S4). Focusing on the detailed behavioral contexts (Table 2), socio-negative interactions such as agonistic interactions and avoidance were predominated by screams, screeches and screech-screams (agonistic interactions: 93%, avoidance: 63%; Figure 6). Thus, a socio-negative context is indicated for these calls. In contrast, in socio-positive interactions such as caravanning, chirps were exclusively produced (caravanning: 100%; Figure 6), indicating their usage in a socio-positive context. A detailed description of the call types including the context can be seen in Table 3.

**Table 2:** Ethogram of behaviors coded during the confrontation experiments of the Etruscan shrews

Behavior	Definition behavior
Avoidance	At least one of the study animals changes its position after the distance between them was reduced, without agonistic behavior having taken place.
Box	At least one of the study animals was inside the housing box.
Caravanning	One animal attaches to the other by biting in the fur left or right to the tail and then they move together.
Combination of behaviors	Combination of *caravanning* and *socio-negative interactions*, with them taking place during the same state event.
Socio-negative interactions	Either one of the study animals chases the other, or a fight occurs between them, or they violently jump away from each other.
Wire mesh	The study animals were in proximity from the different sides of the wire mesh of the dividing wall.
No physical interaction	The study animals were in proximity, but none of the other modifiers took place.

All behaviors were coded when the study animals were within two or fewer stretched body lengths (excluding the tail). A behavior was not coded anew if the animals moved more than two body lengths apart for less than 1 s before returning to proximity. The duration of each behavior event was measured.

**Table 3:** Description of the call types of the Etruscan shrews

Call types	Description	Age group	Context
**Tonal call types**			

Screams	Weakly modulated tonal calls, which were usually long and had an almost constant frequency. They were longer than the rest of the tonal calls and occasionally had a slightly ascending frequency at the beginning for adults and a slightly descending frequency at the end for pups. The screams of the pups had a higher fundamental frequency than the ones of the adults.	Adults/Pups	Socio-negative
Chirps	Short and soft tonal calls. For the adults, the fundamental frequency at the start of the call was low but then it ascended, reaching its peak at the end of the call. For the pups, chirps had a similar contour to the chirps of the adults, or the contour resembled a reversed U, which often had gaps. They were usually produced in bouts.	Adults/Pups	Socio-positive

**Non-tonal call type**			

Screech	Solely non-tonal calls with a tremolo structure. They had a smeared fundamental frequency with numerous pulses.	Adults/Pups	Socio-negative

**Transitional call type**			

Screech – screams	Calls with a non-tonal part similar to the screeches and a tonal part similar to the screams.	Adults/Pups	Socio-negative

Spectrograms of the call types are available in Figure 4.

### Call rates

**Figure 7:**
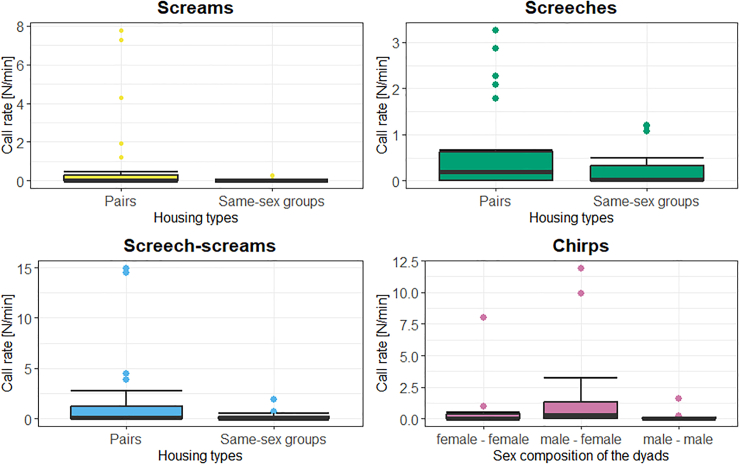
Boxplots illustrating the effects of housing types and sex composition of the dyads on the call rates of the different call types The boxplots represent lower and upper quartiles; the thick black line is the median and the whiskers are the non-outlier range.

As the confronting dyads differed in their sex composition (male-male, female-female, male-female), familiarity (animals knew each other or not), and housing types (animals housed in pairs or in same-sex groups), we investigated the effect of these factors on the call rates using LMEs. The final LMEs contained only the main terms. For screams, screeches, and screech-screams, the call rate was significantly affected by housing type (χ^2^ ≥ 3.83, df = 1, *p* ≤ 0.050; Figure 7), with a higher call rate for animals housed in pairs compared to same-sex groups. Thus, it is suggested that the socio-negative calls are significantly more often produced by animals housed in pairs. In contrast, for chirps, the call rate was significantly affected by the sex composition of the dyad (χ^2^ = 6.95, df = 2, *p* = 0.031; Figure 7), with a higher call rate for the male-female dyads compared to same-sex dyads. Interestingly, a trend toward a significant effect of the housing type on the call rate of chirps was also present (χ^2^ = 3.56, df = 1, *p* = 0.059), with a higher call rate recorded for the animals housed in same-sex groups compared to those housed in pairs. Thus, the socio-positive chirps are significantly more often produced during the confrontation experiments of male-female dyads, with a higher call rate for animals housed in same-sex groups. The detailed results can be found in Table S10 while the mean call rates by call types and influencing factors (sex composition of the dyads, housing type, and familiarity) can be found in Table S11 in the supplementary.

## Discussion

In this study, the first detailed description of the vocal repertoire of Etruscan shrews is delivered. We defined the following call types: screams, screeches (the defensive call type described by Hutterer et al.^55^), screech-screams, and chirps. Thereby, screech-screams constitute a gradation between screeches and screams. All call types were produced by both adults and pups but showed age-related differences in their acoustic parameters. The behavioral context analysis indicated that the chirps were mostly associated with socio-positive contexts, while the rest of the calls were predominantly produced during socio-negative interactions. To conclude, this study demonstrated that gradation in the vocal repertoire of the smallest terrestrial mammal living in a closed habitat is present.

As all call types reported in this study were produced by both adults and pups, it is suggested that the observed call types are innate. The age-related differences reported in the acoustic properties, especially of tonal calls, appear to be related to physical maturation. Smaller pups produced screams with a higher fundamental frequency and a higher mean slope than adult Etruscan shrews, which corresponds to the negative correlation between body size and fundamental frequency reported in most other mammalian species.^21^^,^^24^^,^^25^^,^^58^ This contrasts with findings in piebald and Asian house shrews, where for the majority of call types the fundamental frequency is higher in adults compared to pups or increased with pups’ age.^26^^,^^57^^,^^59^ Interestingly, for chirps, we found no differences in mean and maximum fundamental frequency, but the pups produced chirps with a significantly lower minimum fundamental frequency. Differences were also recorded in the duration and the variability of the contour of the fundamental frequency of the call types, with the chirps and screams of the pups being significantly longer and more variable in their contour than those of the adults (Figure 4). The higher call duration in pups compared to adults contradicts the general observation that with age an increase in lung volume enables animals to produce longer calls.^21^^,^^27^^,^^28^ Therefore, Etruscan shrews may not follow the typical ontogenetic patterns concerning call duration, with the production of longer calls potentially being advantageous for pups, eliciting stronger responses within their social group.^60^ The age-dependent differences in the acoustic properties of screams are evident in the UMAP, where pup screams form a distinct sub-cluster within Cluster 1 (Figure 1). This separation may indicate a gradual change in the acoustic properties of calls as the pups mature. However, despite forming a separate sub-cluster in the UMAP, these calls did not segregate into a distinct cluster even with different settings in the FCM (c = 4, c = 5). This may be a consequence of the acoustic properties used in the analysis, as most of the differing parameters between the screams of pups and adults were fundamental frequency related, which were not available for all calls and thus could not be utilised in the FCM. Concerning the screeches and the screech-screams of the pups, they both had a significantly lower wiener entropy compared to adults, suggesting a higher tonality. As Hutterer et al.^55^ observed that a higher modulation rate in adult screeches of Etruscan shrews results in reduced tonality in the calls, it can be suggested that pup screeches and screech-screams have a slower modulation rate in their tremolo structure compared to adults. This is further supported by the sonogram comparisons of adult and pup screeches and screech-screams in Figure 4, where a lower number of modulations in the calls of Etruscan shrew pups is evident. These observations align with previous findings in piebald shrews,^26^^,^^59^ where the modulation rate increased with pup age. For adult Etruscan shrews, Hutterer^55^ showed that modulation rate is affected by external temperatures, as they have an imperfect thermoregulation.^61^ Since Etruscan shrew pups have little to no fur,^42^ they may lack the necessary core temperature to produce these call types. To determine whether differences in the modulation rate between pups and adults are related to age or differences in thermoregulatory abilities, further studies are needed. In addition, the context necessary to produce screeches and screech-screams may not have been present during the pup vocalization experiments, which could explain the low occurrence of these call types in pups, as adults usually emitted them during agonistic interactions with conspecifics and during the pup vocalization experiments no conspecifics were present.

Etruscan shrews possess a partly graded vocal repertoire since both graded and discrete calls were recorded during this study. This challenges the notion that graded vocal repertoires predominate in species whose habitats allow the usage of visual or contextual signs and have a complex social organisation.^29^^,^^31^^,^^41^ It also raises the question of whether other factors may facilitate the gradation of calls. Previous studies have shown that when gradation is present within a data sample, an optimal number of clusters may not exist.^33^^,^^41^^,^^62^ Thus, it remains unclear whether screech-screams constitute a separate call type or whether the extent of gradation between the two discrete calls contains specific information. Our findings suggest that screeches, screams, and their gradations occur predominantly in socio-negative contexts. Unfortunately, a more detailed description of the behavioral context was not feasible due to the animals’ small size and very fast speed. However, socio-negative interactions can generally be distinguished as aggressive or defensive, with different calls being thereby vocalised.^63^^,^^64^^,^^65^^,^^66^ In addition, dominant individuals can emit different vocalizations compared to the subdominants.^67^ Thus, it is possible that screeches and screams are linked to different dominance statuses or serve varying functions, produced by different animals engaged in a fight. This is consistent with previous observations in Etruscan shrews^42^^,^^53^ and other shrew species.^57^ For example, Fons^42^ reported that in Etruscan shrews during aggressive encounters the individual who wins a fight produces different calls than the individual who lost the fight. Similarly, Schneiderová^57^ noted that during chases Asian house shrews produced squeaks, screams, and short screams, whereas screeches and short screeches were emitted during aggressive encounters. As the dominant Etruscan shrew usually chases the individual that lost the fight, it is expectable that they thereby produce different call types. In addition, it is possible that Etruscan shrews use other sensory signals as contextual signs, such as seismic vibrations, similar to piebald shrews.^59^^,^^68^ Another explanation relates to the anatomical constraints in Etruscan shrews due to their basal hearing and production system. As shrews possess a low number of hair cells, which suggests low frequency resolution,^48^^,^^49^ it is unclear to what extent they perceive the variability present in their calls, as the screeches and the noisy parts of the screech-screams extend over a broad frequency range, encompassing the frequency spectrum of the screams. One further potential explanation for the variability of the calls could be that the vocalizing animals struggle to control their vocal folds, especially during agonistic interactions and high arousal. To investigate whether shrews can acoustically discriminate screams, screeches, and screech-screams combinations, playback experiments are needed, which will further elucidate their function by eliciting different behavioral responses.

Concerning the call rates, we found that almost all calls were produced during the open-door phase when animals were in proximity. The low vocalization rate during the closed-door phase was unexpected, as one might anticipate that Etruscan shrews would produce long-distance calls to establish contact with conspecifics as observed in other mammals.^69^^,^^70^^,^^71^ However, Fons^42^ mentions Etruscan shrews having a reduced visual perception. Instead, evidence from Etruscan shrews’ cortical organization suggests that the most perceived stimuli are tactile stimuli, with 75% of the responsive cortex sites reacting to them.^72^ Tactile communication is facilitated by long whiskers named macrovibrissae, which equip the proboscis of the Etruscan shrews, and by dense, short whiskers named microvibrissae, which surround their mouth.^73^ Trimming these whiskers disrupts prey capture,^74^ while experiments with artificial prey replicas suggest that prey shape alone provides Etruscan shrew with the necessary tactile cues for initiating attacks, even in the absence of other sensory cues.^75^ Based on these short-range prey detection experiments, we know that Etruscan shrews use Gestalt-perception to recognize prey items.^76^ Thus, it is likely that they also use Gestalt to recognize conspecifics outside their nest. Given their lifestyle (living underground) and small size, which makes them vulnerable to predators,^42^ they may refrain from vocalizing to reduce the risk of detection. In addition, since Etruscan shrews are usually aggressive toward unknown individuals,^42^ they may prefer to remain undetected even by their conspecifics. A further possibility is that, due to their high-energy requirements,^42^ vocalizing solely to check for the presence of a conspecific might be too energetically costly. The higher call rate of socio-positive calls (chirps) for the male – female dyads is in accordance with the findings of Schneiderová^57^ concerning *S. murinus*, who underlines that the male courtship call develops from the calls emitted during caravanning, which in both cases are the chirp calls. An interesting finding is also the effect of the housing type of the animals on the call rates, with the call rate of the chirps being higher in animals housed in same sex groups, while the call rate of the socio-negative calls (screams, screeches, and graded calls) was significantly higher in animals housed as pairs. An explanation may be that the animals housed in same sex groups are interested in finding a mating partner, thus being more accepting toward unknown animals. On the other hand, as the animals housed in pairs already have a mating partner, they may be more cautious against unknown animals.

### Limitations of the study

To conclude, this study successfully described the following call types of the Etruscan shrews screeches, screams, chirps, and identified a gradation between two of them (screech-screams). Some of these call types correspond to those reported in the vocal repertoires of other shrew species, such as the Asian house shrew (*Suncus murinus*),^57^ the piebald shrew (*Diplomesodon pulchellum*),^26^ the bicolored white-toothed shrew (*Crocidura leucodon*)^63^ and the long-clawed shrew (*Sorex unguiculatus)*.^77^ In addition, graded calls consisting of both tonal and non-tonal parts were also reported for the Asian house shrew and the piebald shrew.^26^^,^^78^ Nonetheless, most of the vocal repertoires of the aforementioned species are larger, consisting of at least six and up to 17 call types.^26^^,^^57^^,^^77^ Thus, it cannot be ruled out that Etruscan shrews emit additional call types, especially since confrontation experiments can be biased toward more socio-negative behavior. Therefore, recordings in the home cages, especially within the nest, are necessary to showcase the full vocal repertoire of the species. Furthermore, while many shrew species use calls to navigate in novel environments through a simple echo-orientation system,^77^^,^^79^^,^^80^^,^^81^ our study did not report any such calls, or any calls in general when the animals were not in proximity, even though they were in a novel environment. Thus, further studies are necessary to assess whether Etruscan shrews also produce calls for echo-based orientation. In addition, the very small size and fast speed of the animals made it difficult to distinguish the exact behavior the animals demonstrated during the experiments, with the behavioral context analysis being limited to a few categories. Future studies should employ high-speed cameras and a smaller arena to enable a more detailed behavioral analysis. In addition, since shrews vocalise at close distances and within a frequency range that is difficult for human ears to localise, further studies should use acoustic cameras to allow sender allocation.^82^ Finally, playback experiments are needed to clarify the extent to which Etruscan shrews perceive the gradation of socio-negative calls and whether this gradation elicits different behavioral responses, indicating distinct functions.

## Resource availability

### Lead contact

Further information and requests for resources should be directed to and will be fulfilled by the lead contact, Alexandra Langehennig-Peristenidou (Alexandra.Langehennig-Peristenidou@outlook.com).

### Materials availability

This study did not generate new unique reagents.

### Data and code availability

• The raw dataset used in this study is available at the Mendeley repository and is also listed in the key resources table.
• This article does not report the original code.
• All the analyses are described in detail in STAR Methods and any additional information required to reanalyze the data reported in this article is available from the lead contact upon request.

## Acknowledgments

We would like to thank Sönke von den Berg for the technical support, the animal keepers for taking care of the study animals, Yara Silberstein for her assistance during the experiments and the fruitful discussions, Tjard Bergmann for his development of R-scripts for the acoustic analysis and Marleen Lütkemeyer for her assistance in the acoustic analysis of the pup calls. This Open Access publication was funded by the 10.13039/501100001659Deutsche Forschungsgemeinschaft (DFG, German Research Foundation)—491094227 “Open Access Publication Funding” and the 10.13039/501100005629University of Veterinary Medicine Hannover, Foundation.

## Author contributions

Conceptualization, A.L.P. and M.S.; methodology, A.L.P.; data analysis, A.L.P.; resources, F.F.; writing—original draft preparation, A.L.P.; writing—review and editing, M.S., F.F.; visualization, A.L.P.; supervision: M.S. All authors have read and agreed to the published version of the article.

## Declaration of interests

The authors declare no competing interests.

## STAR★Methods

### Key resources table

**Table:** 

REAGENT or RESOURCE	SOURCE	IDENTIFIER
**Deposited data**		

Dataset 1 and Dataset 2 of Etruscan shrew vocal behaviour used in this paper	This paper	Mendeley Data: https://doi.org/10.17632/snxkknzs52.1

**Experimental models: Organisms/strains**		

Etruscan shrews	University of Veterinary Medicine Hannover, animal facility of Institute of Zoology	N/A

**Software and algorithms**		

Praat	Boersma^83^	N/A
GSU Praat Tools	Owren^84^	N/A
BORIS	Friard and Gamba^85^	N/A
R	R_Core_Team^86^	N/A
R Studio	R_Studio_Team^87^	N/A

### Experimental model and study participant details

We tested 33 adult Etruscan shrews (16 males, 17 females; non-lactating and no signs of pregnancy; mean age 9 months, minimum age 2 months, maximum age 19 months) and 12 Etruscan shrew pups (7 males, 5 females; N_litters_ = 6; Litter sizes: 2 – 4 pups), which were born and raised in captivity. For individual identification, the animals were visually marked using veterinary aluminium spray.

At the time of the experiments, the animals were kept in the breeding colony of the Institute of Zoology of the University of Veterinary Medicine Hannover. The animal husbandry fulfilled all recommendations and was approved by the local veterinary authorities (Lower Saxony State Office for Consumer Protection, Food Safety, and Animal Welfare, 42502/1TiHo). The work in this study was conducted in accordance with the European Community regulations on the protection of experimental animals and the guidelines of the German Animal Welfare Act. It was approved by the Niedersächsisches Landesamt für Verbraucherschutz und Lebensmittelsicherheit, Germany (Licence number: 33.8-42502-04-20/3372, date of approval 28 May 2020).

### Method details

#### Animal housing

The adult animals were kept as pairs (one female, one male) or in same sex groups (up to three animals of the same sex and the same litter), while the pups were kept and raised by their parents. The pairs were housed in terrariums made of glass (width x depth x height: 0.6 x 0.4 x 0.4 m), while the same sex groups were accommodated in plastic boxes (width x depth x height: 0.5 x 0.3 x 0.3 m). As bedding served a mixture of dried soil (70 %), sand (15 %) and bark mulch (15 %). The cages were accommodated with various hiding places for the animals while the terrariums of the pairs also included Y-Tong stones with slits to allow nest-building for the breeding. The animals were fed daily with alive Jamaican field crickets (*Gryllus assimilis*). Water was provided *ad libitum*. The animals were accommodated in two air-conditioned rooms (mean temperature: 24°C, relative humidity: 40 – 60 %) with a simulated day/night cycle of 12:12 h (lights on at 07:00).

#### Experimental set-up

The experiments were conducted in a sound-attenuated chamber using an arena of cuboid shape (width x depth x height: 1 x 0.4 x 0.3 m) made of wire mesh. The floor of the arena was a wood panel. The arena was divided into two equal-sized sides by a dividing wall made of wire mesh, allowing the animals to communicate from a distance with each other across different parts of the arena. The dividing wall had an opaque door (diameter 2.5 cm) that, when open, allowed the animals to move from one side of the arena to the other. The vocalisations were recorded using either a Pettersson Ultrasound Detector D1000X (frequency sensitivity: 5 – 235 kHz, Pettersson Elektronik AB, Uppsala, Sweden) or four MKH 8020 microphones (frequency sensitivity: 10 Hz – 70 kHz, Sennheiser electronic GmbH and Co. KG, Wedemark, Germany) connected to a Zoom F4 or F6 Multitrack Field Recorder (K.K. Zoom corporation, Chiyoda, Tokyo, Japan). The audio files were recorded using a sampling frequency of 200 kHz and a 16 bit resolution (Pettersson Ultrasound Detector D1000X) or 192 kHz and a 16 bit resolution (Zoom F4 or F6 Multitrack Field Recorders) and saved as .wav files. Furthermore, the behaviour of the animals was recorded using four Reolink E1 digital cameras (Reolink, Compton, CA, USA) connected to a Synology surveillance system (Synology Inc., New Taipei City, Taiwan). The experimenter was during the experiments outside of the sound-attenuated chamber.

#### Experimental procedure

For the adults, 47 confrontation experiments were conducted. The confrontation experiments consisted of two experimental phases: the closed-door phase, where the animals were alone on the separate sides of the arena, and the open-door phase, where the door was opened and the animals could physically interact with each other. The confronted dyads differed thereby in their sex composition (male – male, female – female, male – female), their familiarity (familiar, unfamiliar) and housing condition (pairs, same sex groups). Each individual was tested three times, confronting (1) a known individual (of the same sex for animals housed in same-sex groups or of the opposite sex for animals housed in pairs), (2) an unknown individual of the same sex and (3) an unknown individual of the opposite sex. To ensure that multiple testing did not affect the results, the order of the different confrontation partners for each individual was randomised by the means of a block randomisation. Each experiment had a total duration of 30 minutes (closed-door phase: 15 minutes, open-door phase: 15 minutes). After the end of the experiments, the animals were brought back to their home cages and released. During the experiments, water and mealworms were available *ad libitum* to the animals and the arena was equipped with houses for them.

For the pups, pup vocalisation experiments were conducted. There, the pups were separated from their parents and siblings and transported to the experimental set-up, where they were exposed to three distinct, consecutive conditions inducing pup calls in previous studies^6^^,^^11^^,^^88^: (1) the pup was placed undisturbed on a paper towel, (2) gentle manipulation by the experimenter, such as poking or turning it on its back and (3) the pup was placed undisturbed on a heat source. Each condition lasted three minutes. After the end of the experiments, the pups were returned to their parents in their home cages and released. The experiments were conducted across three age classes reflecting different developmental stages: 7 – 9 postnatal days (p.d.), 11 – 13 p.d., 15 – 17 p.d.. Each pup was tested in all three age classes, with the exception of two pups which did not participate in the last age class due to health reasons, resulting in a total of 34 pup experiments.

All experiments were conducted at room temperature.

#### Acoustic analysis

For the acoustic analysis, the software Praat^83^ (Version 6.0.42, Phonetic Sciences, University of Amsterdam, Netherlands) and the GSU Praat Tools^84^ 1.9 scripts were used. The measurements were conducted on a call level, with a call being defined as the smallest vocal unit of continuous sound energy for the adults. Since pup calls often showed small gaps in their fundamental frequency, for pups a vocal unit was defined as continuous sound energy with gaps shorter than 50 ms (for how pup calls were defined refer to Figure S5 in the supplementary). Only high-quality calls were measured, which were defined as those with no overlap with other calls or noise and not over-amplified. A total of 22 time-, filter- source- and tonality-related acoustic parameters were measured. The number of available parameters differed between the vocalisations, as for some the fundamental frequency or the formant-like resonance parameters were not measurable. Thus, we had to distinguish tonal calls/elements (= the contour of the fundamental frequency was throughout the call clearly distinguishable and tracked by the Praat algorithm) or non-tonal calls/elements (= the contour of the fundamental frequency was not throughout the call visible or could not automatically be detected). For calls combining both tonal and non-tonal elements, the same parameters as for the non-tonal calls were measured. The formant settings were calculated based on a vocal tract length of 1.2 cm, measured in an Etruscan shrew carcass according to Scheumann et al.^88^ Since pups produced non-tonal calls primarily in the second age class, when they were similar in size to adults, the same formant settings were used for both age groups. As hnr and wiener entropy were measured on a logarithmic scale (dB), we transformed them to a linear scale for all subsequent statistical analyses. An overview of the acoustic parameters can be seen in Table 1, while the settings for the analysis in Praat can be found in Table S12 in the supplementary.

#### Video analysis

Due to the small size and the very fast speed of the animals, it was difficult to distinguish the exact behaviour the animals demonstrated. As hardly any calls were produced during the closed-door phase (N_calls_ = 32), behaviours were solely coded for the open-door phase (N_calls_ = 2130), when the animals were in a proximity of under two body lengths (excluding the tail) of each other. Since animals vocalised only in close proximity and their vocalisations were in the high frequency to ultrasonic range and thus difficult for human ears to localise, it was not possible to reliably assign the calls to the respective sender. Therefore, the behavioural context analysis was limited to a few categories describing dyadic interactions: socio-negative behaviour (e.g., fighting or chasing), avoidance (at least one animal runs away from the other without socio-negative behaviour taking place), caravanning, mixed behaviour (socio-negative and caravanning during the same state event), contact through the wire mesh, at least one animal being inside the box, or no physical interaction (none of the aforementioned behaviours occurred). The ethogram can be seen in Table 2. The behavioural analysis of the video recordings of the confrontation experiments was conducted using BORIS^85^ (Behavioural Observation Research Interactive Software, Version 7.10.5, Torino, Italy). To align the detected vocalisations with the respective behaviours, the onset and offset times from the video analysis were exported as a Praat Text Grid and merged with the TextGrid annotation file of the corresponding audio file.

### Quantification and statistical analysis

For the statistical analysis two datasets were used. Dataset 1 consisted of 1131 high-quality calls (679 adult calls, 452 pup calls) for which acoustic parameters could be measured. These calls were used to define call types based on an unsupervised cluster analysis. Dataset 2 consisted of all the adult Etruscan shrew calls produced during the open-door phase of the confrontation experiments (N_calls_ = 2130), irrespective of their recording quality. For Dataset 2, the calls were visually classified into call types, based on the results of the statistical analysis using Dataset 1.

#### Definition of different call types and impact of age group

To define distinctive call types, we conducted unsupervised cluster analysis in combination with UMAP (Uniform Manifold Approximation and Projection), a dimensionality reduction technique, as a pre-processing step using Dataset 1. This approach offers several advantages, including the automation of the categorisation task without the subjectivity introduced by human observers.^16^ Furthermore, the utilisation of UMAP as a pre-processing step has been suggested to improve the performance of the clustering algorithm, especially since UMAP preserves both the local and global structure of the data.^89^ The UMAP was performed with all acoustic parameters available for all vocalisations of Dataset 1 (see Table 1). To ensure that the different range of measurement units did not bias the analysis, the data was standardised using the z-score standardisation procedure. For the unsupervised cluster analysis, a soft clustering approach similarly as in Wadewitz et al.^62^ was employed, as the visual inspection of the calls implied a gradation. Thus, fuzzy clustering was applied, using the c-means model.^90^ The Fuzzy c-means (FCM) algorithm was iteratively run, with the number of expected clusters predefined based on the visual inspection of the calls and multiple trials with different numbers of clusters (Final settings: number of clusters = 3; maximum number of iterations = 100; degree of fuzzification μ = 2). The FCM differs from the hard clustering approaches, as instead of a solely crisp classification of the calls into different hard clusters it also calculates a membership value for each observation to each cluster. This value represents the degree that each call belongs to a given cluster. Based on the membership values, we calculated typicality coefficients for each call. Typicality coefficients are calculated by subtracting the second largest membership value from the first and is a measure of how representative a call is for the cluster with the highest membership value. The higher the value of the typicality coefficient, the more typical this call is for the given cluster. Based on the typicality coefficients, the typicality and atypicality thresholds were calculated as described by Wadewitz et al.^62^ and Fischer et al.^33^ Based on the typicality and atypicality thresholds, the calls were categorised in different typicality categories according to their typicality coefficients. Calls with typicality coefficients higher than the typicality threshold are considered typical, while calls with typicality coefficients lower than the typicality threshold are considered atypical. Calls with typicality coefficients lower than the typicality threshold but higher than the atypicality threshold are considered neither typical nor atypical (referred to as neither). To test whether typicality coefficients were significantly affected by age group, LMEs were calculated testing the effect of age group (adults, pups) on the typicality coefficients for each cluster (LMEs: typicality coefficients for each cluster ∼ age group, random = ∼1|Individuals, method = 'ML'). The results were illustrated in histograms of the distribution of the typicality coefficients and boxplots of the typicality coefficients for each cluster and age group. For clusters where a gradation was visually identified, linear mixed-effects models (LMEs) were calculated to determine whether differences in voiced percentage across typicality categories (typical, atypical, neither) were significant (LMEs: voiced percentage ∼ typicality categories, random = ∼1|Individuals, method = 'ML'). This analysis aimed to test whether the observed differences in the tonality of the calls could explain the classification of the calls into the different typicality categories (typical, atypical, neither). To further identify which typicality categories differed significantly from each other, least-squares means comparisons were performed, with adjustments made using the Tukey method.

For the clusters for which a gradation was suggested, the effect of tonality-related acoustic parameters (voiced percentage, wiener entropy) on their membership values was investigated. This was achieved through LMEs (LMEs: membership values ∼ tonality-related acoustic parameters, random = ∼1|Individuals, method = 'ML'), while the results were plotted in scatterplots and histograms. Based on the results of the unsupervised cluster analysis and the subsequent analyses concerning the gradation of the calls, different call types for the Etruscan shrews were described, thereby using the nomenclature already established for other shrew species (e.g., Asian house shrew (*Suncus murinus*): Schneiderová,^57^ piebald shrew (*Diplomesodon pulchellum*): Volodin et al.^26^). Graded calls, which combine tonal and non-tonal elements, were treated as a separate call type. Thus, for clusters for where gradation was indicated, the voiced percentages were plotted in histograms to determine boundaries for separating the calls into graded and discrete call types (discrete tonal calls: voiced percentage ≥ 95 %; discrete noisy calls: voiced percentage = 0 %; graded calls: voiced percentage > 0 % and < 95 %). Then, the acoustic parameters available for all calls were compared among the described call types by the means of LMEs (LME: acoustic parameter ∼ described call types, random = ∼1|Individuals, method = 'ML') for the entirety of the calls. Finally, exemplary pairwise comparisons of the acoustic parameters duration, voiced percentage and centre of gravity between the different call types were conducted using least-squares means comparisons, with adjustments made using the Tukey method.

To investigate potential age differences in the described call types, LMEs were calculated. There, we tested for each call type whether the acoustic parameters differed between the adult and pup calls, while controlling for individuals as a random factor (LME: acoustic parameter ∼ age group, random = ∼1|Individuals, method = 'ML'). This approach enabled us to consider also acoustic parameters which were not available for all call types (see Table 1), thus accounting for acoustic characteristics not included in the unsupervised cluster analysis. To control for multiple testing, Fishers Omnibus tests were performed for each call type.^91^

#### Behavioural context analysis

To account for all recorded vocalisations from adult individuals (Dataset 2) and thus provide a more accurate report of the functions of the different call types, all calls were visually classified based on the results of the FCM and the subsequent analyses concerning the tonality of the calls. To ensure consistency, we calculated the level of agreement between the manual classification and the classification of the statistical analysis by the means of a Cohen’s kappa test.^92^ For the Cohen’s kappa test the statistical classification of Dataset 1 was compared to the manual classification of the same calls. Thereby, for the clusters for which gradation was present, the calls were separated into discrete and graded forms as previously described (discrete tonal calls: voiced percentage ≥ 95 %; discrete noisy calls: voiced percentage = 0 %; graded calls: voiced percentage > 0 % and < 95 %).

To investigate whether animals vocalised more frequently in the proximity of conspecifics compared to when they were separated, we calculated the percentage of uttered calls produced in proximity (< 2 body lengths), 1 second before or after the animals were in proximity, as well as for when the animals were not in proximity, for each call type. To investigate the social function of the different call types, we calculated the percentage of the occurrence of each call type per behavioural category in percent. This was done by diving the occurrence of each call type with the total calls produced for each behaviour. In cases where more than one behaviour were coded for a state event (e.g. box and socio-negative interactions), only one was included in this part of the analysis. Actual interactions (e.g. socio-negative interactions, caravanning) were preferred over events relevant to the position of the animals in the arena (box, wire mesh). The behavioural context analysis was conducted only for the adults with the results graphically illustrated in percent bar stacked plots.

#### Call rates

To investigate the effects of sex composition of the dyads, familiarity and housing type on the call rates during the open-door phase, LMEs were calculated using Dataset 2. Call rates were computed for each call type by dividing the number of calls that occurred with the duration of the respective phase for each experiment (N/min). The full models examined the call rate within each call type (test variable) and had the main terms sex composition of the dyad, familiarity, housing type and the interaction terms between them as influencing variables, while controlling for individual 1 and individual 2 of a dyad as random factors (LME: Call_rate_call_type ∼ Sex_composition ∗ Housing_type + Familiarity ∗ Housing_type, random = list(Individual_1 = pdDiag(∼ 1), Individual_2 = pdDiag(∼ 1)) , method = 'ML'). To determine the minimum adequate model (final model), backward stepwise elimination procedure was conducted.^93^ The model selection process involved removing the highest-level interaction term with the highest non-significant p-value at each step. After each elimination, the previous model was compared to the reduced model using Wald test statistics. The elimination procedure continued until (1) only the main terms remained in the model, (2) the remaining interaction terms had significant p-values, or (3) the Wald test statistics revealed a significant difference between the reduced and the previous model. If the final model contained a significant interaction term, the data was split to dissolve the interaction term and new models for the split data sets were run. The models were fitted by maximising the log-likelihood and ANOVA tables with F-tests and p-values were calculated for each model. We only report final models.

All statistical analyses were applied using R^86^ (Version 4.3.3), accessed using the integrated development environment RStudio^87^ (Version 2023.12.1+402); packages: UMAP – “umap” (Version 0.2.10.0); FCM – “e1071” (Version 1.7-14); LMEs – “nlme” (Version 3.1-164), “car” (Version 3.1-2); graphical illustrations – “ggplot2” (Version 3.5.0); Cohen’s kappa test – “psych” (Version 2.4.3).

## References

[1] Bezerra B.M., Souto A. (2008). Structure and usage of the vocal repertoire of *Callithrix jacchus*. Int. J. Primatol..

[2] Gustison M.L., le Roux A., Bergman T.J. (2012). Derived vocalizations of geladas (*Theropithecus gelada*) and the evolution of vocal complexity in primates. Philos. Trans. R. Soc. Lond. B Biol. Sci..

[3] Van Belle S., Estrada A. (2019). The influence of loud calls on intergroup spacing mechanism in black howler monkeys (*Alouatta pigra*). Int. J. Primatol..

[4] Salmi R., Doran-Sheehy D.M. (2014). The function of loud calls (Hoot Series) in wild western gorillas (*Gorilla gorilla*). Am. J. Phys. Anthropol..

[5] Fedurek P., Donnellan E., Slocombe K.E. (2014). Social and ecological correlates of long-distance pant hoot calls in male chimpanzees. Behav. Ecol. Sociobiol..

[6] Silberstein Y., Felmy F., Scheumann M. (2023). Encoding of Arousal and Physical Characteristics in Audible and Ultrasonic Vocalizations of Mongolian Gerbil Pups Testing Common Rules for Mammals. Animals.

[7] Briefer E.F., Aubin T., Mathevon N. (2020). Coding Strategies in Vertebrate Acoustic Communication.

[8] Kulahci I.G., Rubenstein D.I., Ghazanfar A.A. (2015). Lemurs groom-at-a-distance through vocal networks. Anim. Behav..

[9] Langehennig-Peristenidou A., Scheumann M. (2024). Sex differences in the impact of social relationships on individual vocal signatures in grey mouse lemurs (*Microcebus murinus*). Philos. Trans. R. Soc. Lond. B Biol. Sci..

[10] Linn S.N., Boeer M., Scheumann M. (2018). First insights into the vocal repertoire of infant and juvenile Southern white rhinoceros. PLoS One.

[11] Scheumann M., Zimmermann E., Deichsel G. (2007). Context-specific calls signal infants' needs in a strepsirrhine primate, the gray mouse lemur (*Microcebus murinus*). Dev. Psychobiol..

[12] Zaytseva A.S., Volodin I.A., Ilchenko O.G., Volodina E.V. (2020). Audible calls and their ontogenetic relationship with ultrasonic vocalization in a rodent with a wide vocal range, the fat-tailed gerbil (*Pachyuromys duprasi*). Behav. Processes.

[13] Efremova K.O., Volodin I.A., Volodina E.V., Frey R., Lapshina E.N., Soldatova N.V. (2011). Developmental changes of nasal and oral calls in the goitred gazelle *Gazella subgutturosa*, a nonhuman mammal with a sexually dimorphic and descended larynx. Naturwissenschaften.

[14] Takahashi D.Y., Fenley A.R., Teramoto Y., Narayanan D.Z., Borjon J.I., Holmes P., Ghazanfar A.A. (2015). The developmental dynamics of marmoset monkey vocal production. Science.

[15] Branchi I., Santucci D., Alleva E. (2001). Ultrasonic vocalisation emitted by infant rodents: a tool for assessment of neurobehavioural development. Behav. Brain Res..

[16] Langehennig-Peristenidou A., Romero-Mujalli D., Bergmann T., Scheumann M. (2023). Features of animal babbling in the vocal ontogeny of the gray mouse lemur (*Microcebus murinus*). Sci. Rep..

[17] Elowson A.M., Snowdon C.T., Lazaro-Perea C. (1998). Babbling'and social context in infant monkeys: parallels to human infants. Trends Cognit. Sci..

[18] Fernandez A.A., Burchardt L.S., Nagy M., Knörnschild M. (2021). Babbling in a vocal learning bat resembles human infant babbling. Science.

[19] Egnor S.E.R., Hauser M.D. (2004). A paradox in the evolution of primate vocal learning. Trends Neurosci..

[20] ter Haar S.M., Fernandez A.A., Gratier M., Knörnschild M., Levelt C., Moore R.K., Vellema M., Wang X., Oller D.K. (2021). Cross-species parallels in babbling: animals and algorithms. Philos. Trans. R. Soc. Lond. B Biol. Sci..

[21] Fitch W.T., Hauser M.D. (1995). Vocal production in nonhuman primates: Acoustics, physiology, and functional constraints on “honest” advertisement. Am. J. Primatol..

[22] Zhang Y.S., Takahashi D.Y., Liao D.A., Ghazanfar A.A., Elemans C.P.H. (2019). Vocal state change through laryngeal development. Nat. Commun..

[23] Scheumann M., Linn S., Zimmermann E. (2017). Vocal greeting during mother-infant reunions in a nocturnal primate, the gray mouse lemur (*Microcebus murinus*). Sci. Rep..

[24] Khan C.B., Markowitz H., McCowan B. (2006). Vocal development in captive harbor seal pups, *Phoca vitulina richardii*: Age, sex, and individual differencesa). J. Acoust. Soc. Am..

[25] Gouzoules H., Gouzoules S. (1989). Design features and developmental modification of pigtail macaque, *Macaca nemestrina*, agonistic screams. Anim. Behav..

[26] Volodin I.A., Zaytseva A.S., Ilchenko O.G., Volodina E.V. (2015). Small mammals ignore common rules: a comparison of vocal repertoires and the acoustics between pup and adult piebald shrews *Diplomesodon pulchellum*. Ethology.

[27] Zhang Y.S., Ghazanfar A.A. (2018). Vocal development through morphological computation. PLoS Biol..

[28] Gultekin Y.B., Hildebrand D.G.C., Hammerschmidt K., Hage S.R. (2021). High plasticity in marmoset monkey vocal development from infancy to adulthood. Sci. Adv..

[29] Gros-Louis J.J., Perry S.E., Fichtel C., Wikberg E., Gilkenson H., Wofsy S., Fuentes A. (2008). Vocal repertoire of *Cebus capucinus*: acoustic structure, context, and usage. Int. J. Primatol..

[30] Marler P., Bateson P.P.G., Hinde R.A. (1976). Growing points in ethology..

[31] Keenan S., Lemasson A., Zuberbühler K. (2013). Graded or discrete? A quantitative analysis of Campbell's monkey alarm calls. Anim. Behav..

[32] Hammerschmidt K., Fischer J. (1998). The vocal repertoire of Barbary macaques: a quantitative analysis of a graded signal system. Ethology.

[33] Fischer J., Wadewitz P., Hammerschmidt K. (2017). Structural variability and communicative complexity in acoustic communication. Anim. Behav..

[34] Fischer J., Hammerschmidt K., Cheney D.L., Seyfarth R.M. (2001). Acoustic Features of Female Chacma Baboon Barks. Ethology.

[35] McComb K., Semple S. (2005). Coevolution of vocal communication and sociality in primates. Biol. Lett..

[36] Bouchet H., Blois-Heulin C., Lemasson A. (2013). Social complexity parallels vocal complexity: a comparison of three non-human primate species. Front. Psychol..

[37] Manser M.B., Jansen D.A.W.A.M., Graw B., Hollén L.I., Bousquet C.A.H., Furrer R.D., le Roux A., Naguib M., Barrett L., Brockmann H.J., Healy S., Mitani J.C., Roper T.J., Simmons L.W. (2014). Advances in the Study of Behavior.

[38] Pollard K.A., Blumstein D.T. (2012). Evolving communicative complexity: insights from rodents and beyond. Philos. Trans. R. Soc. Lond. B Biol. Sci..

[39] Freeberg T.M., Dunbar R.I.M., Ord T.J. (2012). Social complexity as a proximate and ultimate factor in communicative complexity. Philos. Trans. R. Soc. Lond. B Biol. Sci..

[40] Lima S.G.C., Sousa-Lima R.S., Tokumaru R.S., Nogueira-Filho S.L.G., Nogueira S.S.C. (2018). Vocal complexity and sociality in spotted paca (*Cuniculus paca*). PLoS One.

[41] Cusano D.A., Noad M.J., Dunlop R.A. (2021). Fuzzy clustering as a tool to differentiate between discrete and graded call types. JASA Express Lett..

[42] Fons R. (1974).

[43] Geyer B., Erickson N.A., Müller K., Grübel S., Hueber B., Hetz S.K., Brecht M. (2022). Establishing and maintaining an Etruscan Shrew colony. J. Am. Assoc. Lab. Anim. Sci..

[44] Berg M. (2016). A miniscule model for research. Lab Anim..

[45] Zacher A.C., Felmy F. (2024). Anatomy of superior olivary complex and lateral lemniscus in Etruscan shrew. Sci. Rep..

[46] Alonso-Nanclares L., Rodríguez J.R., Merchan-Perez A., González-Soriano J., Plaza-Alonso S., Cano-Astorga N., Naumann R.K., Brecht M., DeFelipe J. (2023). Cortical synapses of the world's smallest mammal: An FIB/SEM study in the Etruscan shrew. J. Comp. Neurol..

[47] Naumann R.K. (2015). Even the Smallest Mammalian Brain Has Yet to Reveal Its Secrets. Brain Behav. Evol..

[48] Hunt R.M., Korth W.W. (1980). The auditory region of dermoptera: Morphology and function relative to other living mammals. J. Morphol..

[49] Fleischer G., Fleischer G. (1978). Evolutionary Principles of the Mammalian Middle Ear.

[50] Jürgens K.D. (2002). Etruscan shrew muscle: the consequences of being small. J. Exp. Biol..

[51] Chen Z.-Z., He K., Huang C., Wan T., Lin L.-K., Liu S.-Y., Jiang X.-L. (2017). Integrative systematic analyses of the genus *Chodsigoa* (Mammalia: Eulipotyphla: Soricidae), with descriptions of new species. Zool. J. Linn. Soc..

[52] Vogel P. (1974). Kälteresistenz und reversible Hypothermie der Etruskerspitzmaus. Z. Säugetierkunde.

[53] Spitzenberger F. (1990). *Suncus etruscus* (Savi, 1822)–Etruskerspitzmaus. Handb. Säugetiere Eur..

[54] Vogel P. (1969). Biologische Beobachtungen an Etruskerspitzmäusen. Z. Säugetierkunde.

[55] Hutterer R., Vogel P., Frey H., Genoud M. (1979). Vocalization of the shrews *Suncus etruscus* and *Crocidura russula* during normothermia and torpor. Acta Theriol..

[56] Hutterer R. (1978). Paarungsrufe der Wasserspitzmaus (*Neomys fodiens*) und verwandte Laute weiterer Soricidae. Z Säugetierk.

[57] Schneiderová I. (2014). Vocal repertoire ontogeny of the captive Asian house shrew *Suncus murinus* suggests that the male courtship call develops from the caravanning call of the young. Acta Theriol..

[58] Bowling D.L., Garcia M., Dunn J.C., Ruprecht R., Stewart A., Frommolt K.H., Fitch W.T. (2017). Body size and vocalization in primates and carnivores. Sci. Rep..

[59] Zaytseva A.S., Volodin I.A., Mason M.J., Frey R., Fritsch G., Ilchenko O.G., Volodina E.V. (2015). Vocal development during postnatal growth and ear morphology in a shrew that generates seismic vibrations, *Diplomesodon pulchellum*. Behav. Processes.

[60] Blumstein D.T., Richardson D.T., Cooley L., Winternitz J., Daniel J.C. (2008). The structure, meaning and function of yellow-bellied marmot pup screams. Anim. Behav..

[61] Fons R., Sender S., Peters T., Jürgens K.D. (1997). Rates of Rewarming, Heart and Respiratory Rates and Their Significance for Oxygen Transport During Arousal From Torpor in the Smallest Mammal, the Etruscan Shrew *Suncus Etruscus*. J. Exp. Biol..

[62] Wadewitz P., Hammerschmidt K., Battaglia D., Witt A., Wolf F., Fischer J. (2015). Characterizing Vocal Repertoires—Hard vs. Soft Classification Approaches. PLoS One.

[63] Simeonovska-Nikolova D.M. (2004). Vocal communication in the bicoloured white-toothed shrew *Crocidura leucodon*. Acta Theriol..

[64] Rieger N.S., Marler C.A. (2018). The function of ultrasonic vocalizations during territorial defence by pair-bonded male and female California mice. Anim. Behav..

[65] Searcy Y.M., Caine N.G. (2003). Hawk calls elicit alarm and defensive reactions in captive Geoffroy’s marmosets (*Callithrix geoffroyi*). Folia Primatol..

[66] Schwartz J.J. (1989). Graded Aggressive Calls of the Spring Peeper, *Pseudacris crucifer*. Herpetologica.

[67] Kitchen D.M., Seyfarth R.M., Fischer J., Cheney D.L. (2003). Loud calls as indicators of dominance in male baboons (*Papio cynocephalus ursinus*). Behav. Ecol. Sociobiol..

[68] Volodin I.A., Zaytseva A.S., Ilchenko O.G., Volodina E.V., Chebotareva A.L. (2012). Measuring airborne components of seismic body vibrations in a Middle-Asian sand-dwelling Insectivora species, the piebald shrew (*Diplomesodon pulchellum*). J. Exp. Biol..

[69] Stein R.M., Rachlow J.L. (2023). Acoustic ecology of terrestrial mammals: a new Signaller–Receiver Conceptual Framework. Mamm Rev..

[70] Ferreira L.S., Sábato V., Pinheiro T.A., Neto E., Rocha L.H., Baumgarten J., Rodrigues F.H., Sousa-Lima R.S. (2022). Long-Distance Counter Calling in Maned Wolves: Friends or Foes?. Animals.

[71] Klenova A.V., Chelysheva E.V., Vasilieva N.A., Volodin I.A., Volodina E.V. (2024). Acoustic features of long-distance calls of wild cheetahs (*Acinonyx jubatus*) are linked to the caller age from newborns to adults. Ethology.

[72] Roth-Alpermann C., Anjum F., Naumann R., Brecht M. (2010). Cortical organization in the Etruscan shrew (*Suncus etruscus*). J. Neurophysiol..

[73] Roth-Alpermann C., Brecht M., Prescott T., Ahissar E., Izhikevich E. (2016). Scholarpedia of Touch.

[74] Anjum F., Brecht M. (2012). Tactile experience shapes prey-capture behavior in Etruscan shrews. Front. Behav. Neurosci..

[75] Anjum F., Turni H., Mulder P.G.H., van der Burg J., Brecht M. (2006). Tactile guidance of prey capture in Etruscan shrews. Proc. Natl. Acad. Sci. USA.

[76] Brecht M., Naumann R., Anjum F., Wolfe J., Munz M., Mende C., Roth-Alpermann C. (2011). The neurobiology of Etruscan shrew active touch. Philos. Trans. R. Soc. Lond. B Biol. Sci..

[77] Sanchez L., Ohdachi S.D., Kawahara A., Echenique-Diaz L.M., Maruyama S., Kawata M. (2019). Acoustic emissions of *Sorex unguiculatus* (Mammalia: Soricidae): Assessing the echo-based orientation hypothesis. Ecol. Evol..

[78] Schneiderová I., Zouhar J. (2014). Resting-Associated Vocalization Emitted by Captive Asian House Shrews (*Suncus murinus*): Acoustic Structure and Variability in an Unusual Mammalian Vocalization. PLoS One.

[79] von Merten S., Siemers B.M. (2020). Shrew twittering call rate is high in novel environments—a lab-study. Mamm. Res..

[80] Siemers B.M., Schauermann G., Turni H., von Merten S. (2009). Why do shrews twitter? Communication or simple echo-based orientation. Biol. Lett..

[81] Zsebők S., Czabán D., Farkas J., Siemers B.M., von Merten S. (2015). Acoustic species identification of shrews: Twittering calls for monitoring. Ecol. Inf..

[82] Matsumoto J., Kanno K., Kato M., Nishimaru H., Setogawa T., Chinzorig C., Shibata T., Nishijo H. (2022). Acoustic camera system for measuring ultrasound communication in mice. iScience.

[83] Boersma P. (2001). Praat, a system for doing phonetics by computer. Glot. Int..

[84] Owren M.J. (2008). GSU Praat Tools: Scripts for modifying and analyzing sounds using Praat acoustics software. Behav. Res. Methods.

[85] Friard O., Gamba M. (2016). BORIS: a free, versatile open-source event-logging software for video/audio coding and live observations. Methods Ecol. Evol..

[86] R_Core_Team (2020).

[87] R_Studio_Team (2021). RStudio: integrated development environment for R.

[88] Scheumann M., Roser A.-E., Konerding W., Bleich E., Hedrich H.-J., Zimmermann E. (2012). Vocal correlates of sender-identity and arousal in the isolation calls of domestic kitten (*Felis silvestris catus*). Front. Zool..

[89] Allaoui M., Kherfi M.L., Cheriet A., El Moataz A., Mammass D., Mansouri A., Nouboud F. (2020). Image and Signal Processing.

[90] Zadeh L.A. (1965). Fuzzy sets. Inf. Control.

[91] Haccou P., Meelis E. (1992).

[92] Landis J.R., Koch G.G. (1977). An Application of Hierarchical Kappa-type Statistics in the Assessment of Majority Agreement among Multiple Observers. Biometrics.

[93] Zuur A.F., Ieno E.N., Walker N.J., Saveliev A.A., Smith G.M. (2009).

