# Supplementary material for: Graded calls of the smallest terrestrial mammal, the Etruscan shrew, living in a closed habitat
Source: iScience. 2024 Nov 1;27(12):111297. doi: 10.1016/j.isci.2024.111297 (PMC11612789; doi:10.1016/j.isci.2024.111297)
Supplement: Document S1. Figures S1–S5 and Tables S1–S12 [file mmc1.pdf]

iScience, Volume *27*

Supplemental information

Graded calls of the smallest terrestrial mammal, the Etruscan shrew, living in a closed habitat

Alexandra Langehennig-Peristenidou, Felix Felmy, and Marina Scheumann

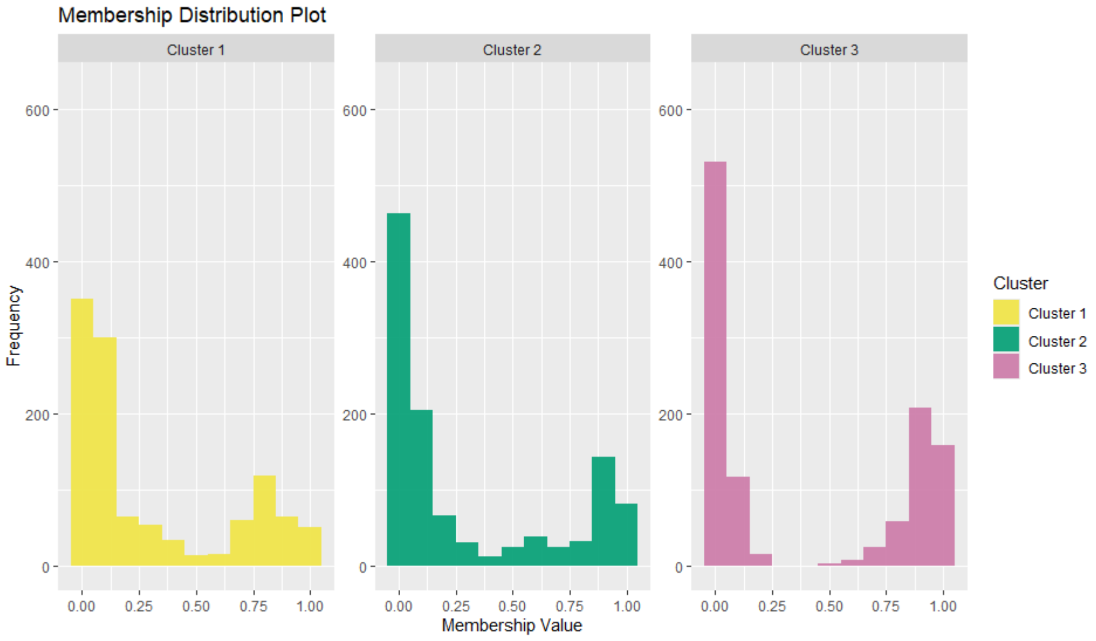

Fig. S1 Distribution plots for the membership values for each cluster acquired by the FCM.

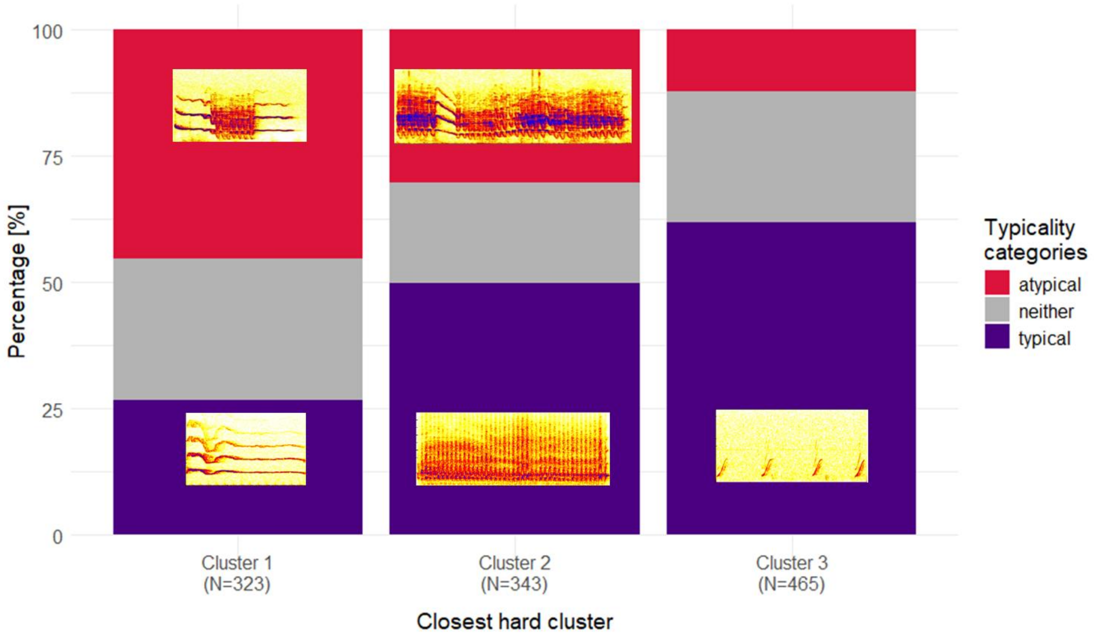

Fig. S2 Proportions of typicality categories for the clusters acquired by the FCM.

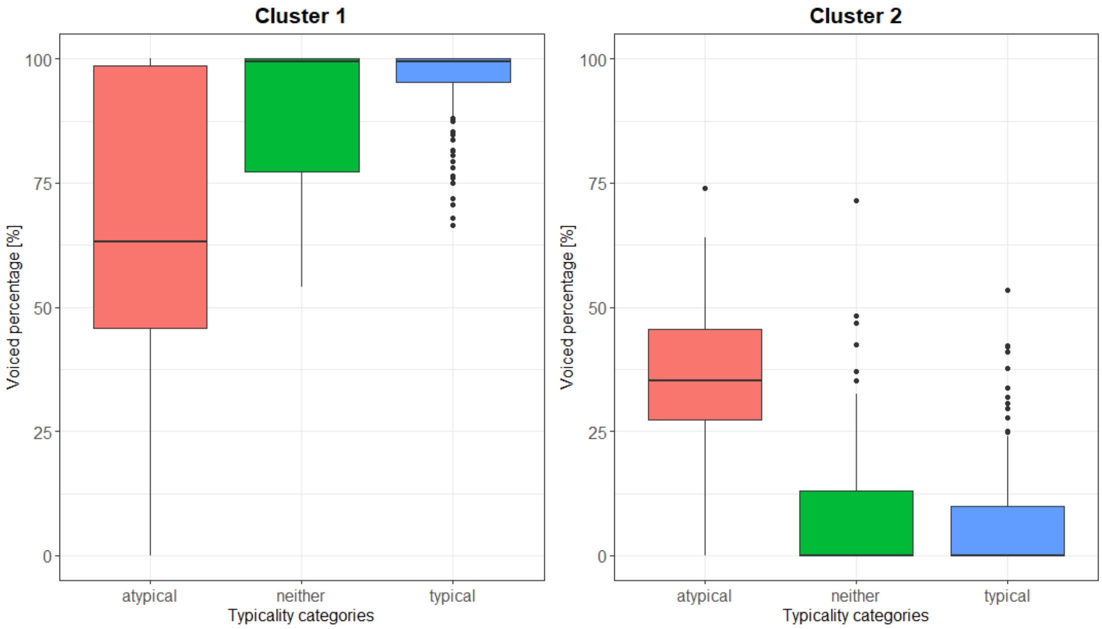

Fig. S3 Distribution of the typicality categories in relation to the voiced percentage for Cluster 1 and Cluster 2. The boxplots represent lower and upper quartiles; thick black line is the median and whiskers are the non-outlier range.

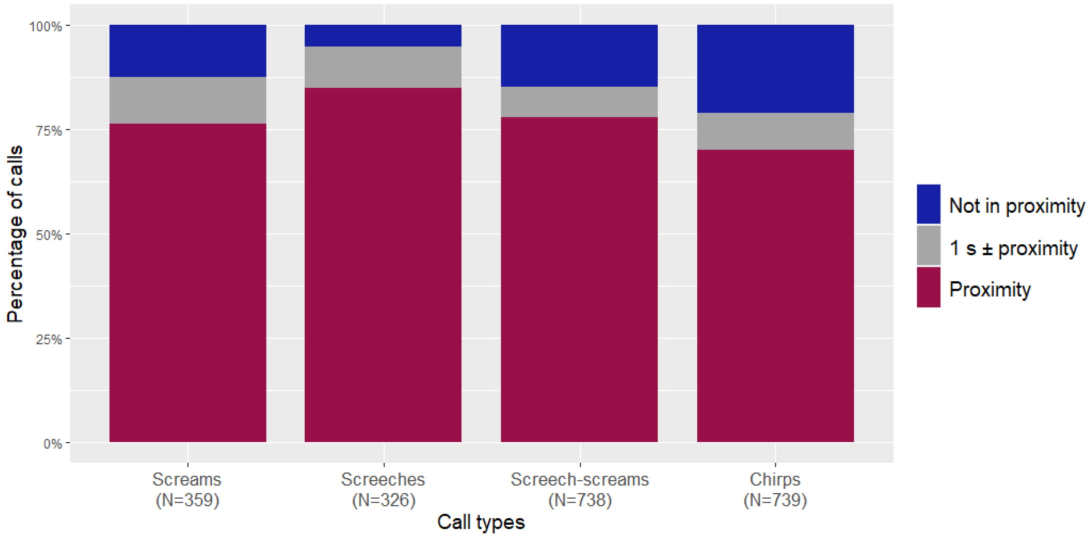

Fig. S4 Percentage of calls produced in the different proximity measures for each call type defined in this study. N: number of calls for each cluster.

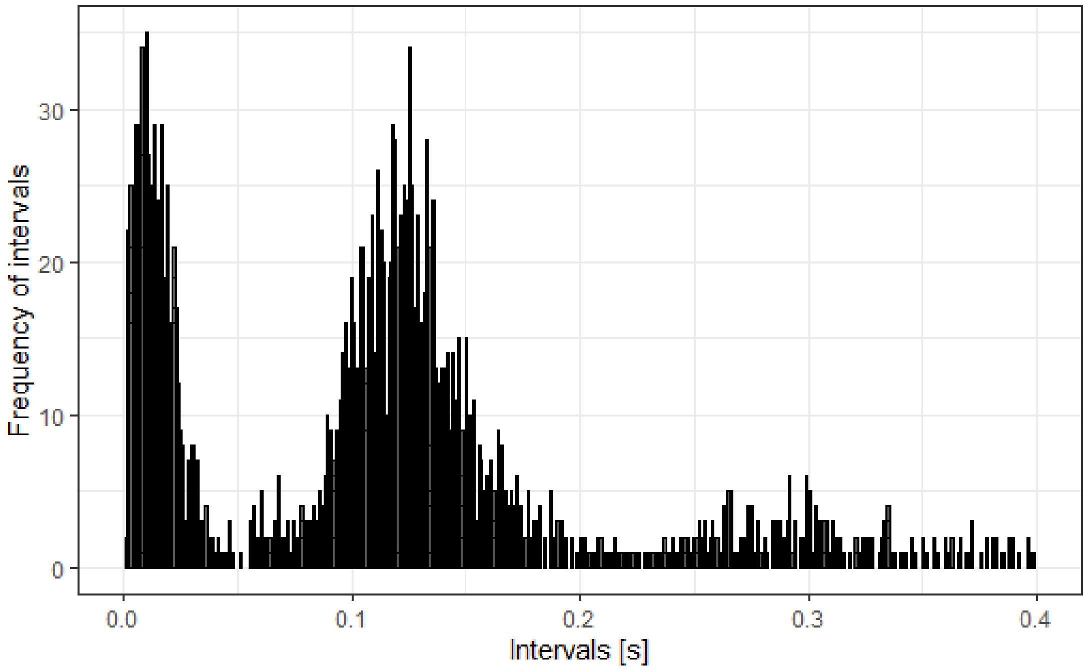

Fig. S5 Histogram plotting the individual intervals between the vocal units of continuous sound energy in two audio files of Etruscan shrew pups.

To differentiate between gaps within pup calls and inter-call intervals between calls, we plotted the intervals between the offset of a vocal unit and the onset of the following vocal unit. The plotted data showed a bimodal distribution, with the first peak indicating the gaps within the calls and the second peak indicating the gaps between the calls. Based on this, a call was defined as vocal unit with gaps no longer than 0.05 s. Note that for better visualisation, the gaps longer than 0.4 s were omitted for the histogram (326 values).

Tab. S1 Acoustic description of the clusters acquired by the FCM. SD = standard deviation; NA = not available. For Cluster 1 and Cluster 3 the tonal parameters were calculated only for the subset of each cluster for which these parameters were available.

	**Cluster 1 (N_{calls} = 323)**		**Cluster 2 (N_{calls} = 343)**		**Cluster 3 (N_{calls} = 465)**	
	**Mean**	**SD**	**Mean**	**SD**	**Mean**	**SD**
Duration [s]	0.16	0.07	0.13	0.08	0.03	0.02
Time of peak amplitude [s]	0.04	0.05	0.05	0.06	0.02	0.01
Voiced percentage [%]	81.01	23.48	15.16	18.37	97.35	6.89
Harmonics-to-noise ratio	71.73	195.42	7.51	3.94	15.36	11.47
Centre of gravity [kHz]	25.83	3.75	23.57	3.03	19.74	4.45
Standard deviation [kHz]	5.80	1.62	7.55	1.96	8.22	2.37
Skewness	1.91	1.49	0.87	0.85	3.02	1.76
Kurtosis	18.49	17.68	7.46	7.20	23.27	22.35
Wiener entropy	0.48	0.10	0.80	0.12	0.76	0.13

Tab. S2 Percentages of calls classified as typical, neither or atypical for each cluster acquired by the FCM. Neither refers to calls which were categorised as neither typical nor atypical.

Cluster	Typical	Neither	Atypical
Cluster 1 (N_{calls} = 323)	26.63 %	28.17 %	45.20 %
Cluster 2 (N_{calls} = 343)	49.85 %	19.83 %	30.32 %
Cluster 3 (N_{calls} = 465)	61.94 %	25.81 %	12.26 %
Sum (N_{calls} = 1131)	48.19 %	24.67 %	27.14 %

Tab. S3 Results of the LME models testing for age differences in the typicality coefficients for each cluster. Bold p-values represent a significant difference $p < 0.05$, df = degrees of freedom; se = standard error.

Cluster	Estimate	se	df	t-value	p-value
Cluster 1	0.03	0.05	9	0.57	0.586
Cluster 2	-0.11	0.10	26	-1.06	0.300
Cluster 3	0.09	0.05	22	1.80	0.085

Tab. S4 Results of the LME models testing the relationship between membership values to Cluster 1 and Cluster 2 and the tonality-related acoustic parameters voiced percentage and wiener entropy. Bold p-values represent a significant difference p < 0.05, df = degrees of freedom; se = standard error.

Cluster	Tonality-related acoustic parameter	Estimate	se	df	t-value	p-value
Cluster 1	Voiced percentage	0.01	0.00	636	52.78	**<0.001**
Cluster 1	Wiener entropy	-1.69	0.04	636	-42.38	**<0.001**
Cluster 2	Voiced percentage	-0.01	0.00	636	-56.42	**<0.001**
Cluster 2	Wiener entropy	1.80	0.04	636	45.70	**<0.001**

Tab. S5 Acoustic description of the call types described in this study. SD = standard deviation; NA = not available.

	**Screams** (**N_{calls} = 173**)		**Screeches** (**N_{calls} = 171**)		**Screech-screams** (**N_{calls} = 322**)		**Chirps** (**N_{calls} = 465**)	
	**Mean**	**SD**	**Mean**	**SD**	Mean	SD	**Mean**	**SD**
Duration [s]	0.16	0.07	0.11	0.08	0.15	0.06	0.03	0.02
Time of peak amplitude [s]	0.04	0.06	0.04	0.05	0.04	0.05	0.02	0.01
Voiced percentage [%]	99.31	1.12	0.00	0.00	44.05	22.34	97.35	6.89
Harmonics-to-noise ratio	100.46	226.46	5.22	3.46	23.20	100.41	15.36	11.47
Centre of gravity [kHz]	26.78	4.47	22.94	3.63	24.44	2.20	19.74	4.45
Standard deviation [kHz]	5.73	1.77	8.02	1.85	6.53	1.83	8.22	2.37
Skewness	1.70	1.67	0.83	0.89	1.49	1.18	3.02	1.76
Kurtosis	20.91	21.45	6.06	6.73	12.04	10.06	23.27	22.35
Wiener entropy	0.42	0.06	0.87	0.10	0.65	0.12	0.76	0.13
Minimum fundamental frequency [kHz]	19.55	5.66	NA	NA	NA	NA	11.69	4.25
Time of minimum fundamental frequency [s]	0.14	0.08	NA	NA	NA	NA	0.01	0.02
Maximum fundamental frequency [kHz]	30.95	6.17	NA	NA	NA	NA	28.09	5.65
Time of maximum fundamental frequency [s]	0.02	0.03	NA	NA	NA	NA	0.02	0.01
Mean fundamental frequency [kHz]	24.49	5.92	NA	NA	NA	NA	19.87	4.41
Standard deviation of the fundamental frequency [kHz]	2.47	0.92	NA	NA	NA	NA	5.54	2.31
Mean slope [kHz/s]	372.04	202.96	NA	NA	NA	NA	999.34	429.42
Frequency of first formant [kHz]	NA	NA	17.05	3.05	19.13	3.00	NA	NA
Bandwidth of first formant [kHz]	NA	NA	5.57	4.79	2.81	5.56	NA	NA
Frequency of second formant [kHz]	NA	NA	26.21	2.99	27.32	4.13	NA	NA
Bandwidth of second formant [kHz]	NA	NA	5.27	5.44	8.25	9.05	NA	NA

	**Screams**<br><br>**(N_{calls} = 173)**		**Screeches**<br><br>**(N_{calls} = 171)**		**Screech-screams**<br>**(N_{calls} = 322)**		**Chirps**<br><br>**(N_{calls} = 465)**	
	**Mean**	**SD**	**Mean**	**SD**	Mean	SD	**Mean**	**SD**
Frequency of third formant [kHz]	NA	NA	37.37	3.69	39.57	4.37	NA	NA
Bandwidth of third formant [kHz]	NA	NA	7.63	9.22	4.85	8.23	NA	NA

Tab. S6 Results of the LME models testing whether the acoustic parameters available for all calls differ significantly among the described call types. Bold p-values represent a significant effect p < 0.05. df = degrees of freedom.

Predictors	χ^2	df	p-value
**All calls**			
Duration [s]	349.78	3	**< 0.001**
Time of peak amplitude [s]	72.32	3	**< 0.001**
Voiced percentage [%]	6894.80	3	**< 0.001**
Harmonics-to-noise ratio	21.28	3	**< 0.001**
Centre of gravity [kHz]	113.09	3	**< 0.001**
Standard deviation [kHz]	24.15	3	**< 0.001**
Skewness	102.64	3	**< 0.001**
Kurtosis	110.53	3	**< 0.001**
Wiener entropy	835.59	3	**< 0.001**

Tab. S7 Pairwise comparisons of the acoustic parameters duration, voiced percentage and centre of gravity between the different call types. Bold: $p < 0.05$; df = degree of freedom; SE = Standard error.

Contrast			Estimate	SE	t – ratio	p – value
Duration (df = 1091)						
Chirp	-	Screech-scream	-0.12	0.01	-18.51	**< 0.001**
Chirp	-	Scream	-0.11	0.01	-15.22	**< 0.001**
Chirp	-	Screech	-0.09	0.01	-12.69	**< 0.001**
Screech-scream	-	Scream	0.01	0.01	1.96	0.203
Screech-scream	-	Screech	0.03	0.01	5.86	**< 0.001**
Scream	-	Screech	0.02	0.01	3.53	**0.002**
Voiced percentage (df = 1091)						
Chirp	-	Screech-scream	56.46	1.34	42.26	**< 0.001**
Chirp	-	Scream	2.29	1.52	1.51	0.433
Chirp	-	Screech	98.13	1.41	69.62	**< 0.001**
Screech-scream	-	Scream	-54.16	1.22	-44.34	**< 0.001**
Screech-scream	-	Screech	41.68	1.32	31.69	**< 0.001**
Scream	-	Screech	95.84	1.51	63.32	**< 0.001**
Centre of gravity (df = 1091)						
Chirp	-	Screech-scream	-3.83	0.42	-9.04	**< 0.001**
Chirp	-	Scream	-4.92	0.47	-10.47	**< 0.001**
Chirp	-	Screech	-3.07	0.45	-6.81	**< 0.001**
Screech-scream	-	Scream	-1.09	0.32	-3.44	**0.003**
Screech-scream	-	Screech	0.76	0.36	2.13	0.145
Scream	-	Screech	1.85	0.42	4.44	**< 0.001**

Tab. S8 Results of the LME models testing whether significant differences in the acoustic parameters of the described call types are present between adults and pups. Bold p-values represent a significant difference $p < 0.05$. df = degrees of freedom; se = standard error. Positive values: pups > adults. negative values: pups < adults.

Acoustic parameter	Estimate	se	df	t-value	p-value
**Screams (N_{adults} = 120, N_{pups} = 53)**					
Duration [s]	0.11	0.02	7	4.66	**0.002**
Time of peak amplitude [s]	0.08	0.01	7	10.05	**<0.001**
Voiced percentage [%]	0.08	0.19	7	0.44	0.673
Harmonics-to-noise ratio	214.64	85.58	7	2.51	**0.041**
Centre of gravity [kHz]	7.88	1.79	7	4.39	**0.003**
Standard deviation [kHz]	0.25	0.71	7	0.36	0.732
Skewness	-0.97	0.65	7	-1.49	0.181
Kurtosis	16.10	9.63	7	1.67	0.139
Wiener entropy	-0.04	0.01	7	-3.40	**0.011**
Minimum fundamental frequency [kHz]	7.03	3.12	7	2.25	0.059
Time of minimum fundamental frequency [s]	0.12	0.03	7	4.41	**0.003**
Maximum fundamental frequency [kHz]	12.27	2.18	7	5.63	**0.001**
Time of maximum fundamental frequency [s]	0.02	0.01	7	1.95	0.093
Mean fundamental frequency [kHz]	10.91	2.16	7	5.04	**0.002**
Standard deviation of the fundamental frequency [kHz]	0.77	0.61	7	1.26	0.249
Mean slope [kHz/s]	305.44	86.51	7	3.53	**0.010**
**Screeches (N_{adults} = 164, N_{pups} = 7)**					
Duration [s]	-0.03	0.04	22	-0.75	0.460
Time of peak amplitude [s]	-0.01	0.02	22	-0.48	0.635
Harmonics-to-noise ratio	5.74	1.41	22	4.07	**0.001**
Centre of gravity [kHz]	-0.79	1.78	22	-0.44	0.663
Standard deviation [kHz]	-0.61	1.13	22	-0.54	0.592
Skewness	1.22	0.43	22	2.84	**0.010**
Kurtosis	4.59	2.97	22	1.55	0.136
Wiener entropy	-0.18	0.04	22	-4.38	**<0.001**
Frequency of first formant [kHz]	0.88	1.40	22	0.63	0.536
Bandwidth of first formant [kHz]	-1.48	1.86	22	-0.79	0.436
Frequency of second formant [kHz]	1.24	1.34	22	0.93	0.364
Bandwidth of second formant [kHz]	2.06	2.10	22	0.98	0.339
Frequency of third formant [kHz]	1.16	1.59	22	0.73	0.474
Bandwidth of third formant [kHz]	1.89	3.57	22	0.53	0.601
**Screech-screams (N_{adults} = 307, N_{pups} = 15)**					
Duration [s]	0.02	0.02	19	1.32	0.203
Time of peak amplitude [s]	-0.02	0.02	19	-0.95	0.355
Voiced percentage [%]	21.75	9.19	19	2.37	**0.029**
Harmonics-to-noise ratio	187.15	38.17	19	4.90	**<0.001**
Centre of gravity [kHz]	5.12	1.46	19	3.51	**0.002**

Acoustic parameter	Estimate	se	df	t-value	p-value
Standard deviation [kHz]	-1.86	1.01	19	-1.83	0.083
Skewness	0.19	0.48	19	0.40	0.693
Kurtosis	13.89	4.17	19	3.33	**0.004**
Wiener entropy	-0.17	0.05	19	-3.23	**0.004**
Frequency of first formant [kHz]	-0.10	1.39	19	-0.07	0.946
Bandwidth of first formant [kHz]	0.10	1.71	19	0.06	0.954
Frequency of second formant [kHz]	1.20	1.27	19	0.95	0.355
Bandwidth of second formant [kHz]	-3.05	3.02	19	-1.01	0.325
Frequency of third formant [kHz]	1.15	1.88	19	0.61	0.547
Bandwidth of third formant [kHz]	-0.05	2.73	19	-0.02	0.986
**Chirps (N_{adults} = 88, N_{pups} = 377)**					
Duration [s]	0.01	0.00	22	3.20	**0.004**
Time of peak amplitude [s]	0.01	0.00	22	3.86	**0.001**
Voiced percentage [%]	-1.56	1.15	22	-1.35	0.190
Harmonics-to-noise ratio	3.65	2.37	22	1.54	0.138
Centre of gravity [kHz]	-0.58	1.46	22	-0.40	0.694
Standard deviation [kHz]	-2.62	1.36	22	-1.93	0.067
Skewness	0.79	0.46	22	1.73	0.098
Kurtosis	0.47	5.20	22	0.09	0.929
Wiener entropy	-0.03	0.05	22	-0.53	0.603
Minimum fundamental frequency [kHz]	-4.48	1.71	22	-2.62	**0.016**
Time of minimum fundamental frequency [s]	0.00	0.00	22	-0.24	0.816
Maximum fundamental frequency [kHz]	2.96	1.56	22	1.90	0.071
Time of maximum fundamental frequency [s]	0.01	0.00	22	6.62	**<0.001**
Mean fundamental frequency [kHz]	-0.61	1.42	22	-0.43	0.671
Standard deviation of the fundamental frequency [kHz]	2.29	0.68	22	3.34	**0.003**
Mean slope [kHz/s]	-157.52	202.28	22	-0.78	0.444

Tab. S9 Acoustic description of the call types described in this study for adults and pups separately. SD = standard deviation; NA = not available.

ADULTS								
	Screams (N_{calls} = 120)		Screeches (N_{calls} = 164)		Screech-Screams (N_{calls} = 307)		Chirps (N_{calls} = 88)	
	**Mean**	**SD**	**Mean**	**SD**	**Mean**	**SD**	**Mean**	**SD**
Duration [s]	0.13	0.05	0.11	0.09	0.15	0.06	0.02	0.02
Time of peak amplitude [s]	0.02	0.03	0.04	0.05	0.04	0.05	0.01	0.01
Voiced percentage [%]	99.29	1.25	0.00	0.00	43.40	21.74	99.00	4.66
Harmonics-to-noise ratio	22.76	3.28	4.98	2.54	12.71	4.59	11.87	7.19
Centre of gravity [kHz]	24.56	2.13	22.97	3.66	24.25	1.81	19.24	5.42
Standard deviation [kHz]	5.52	1.52	8.03	1.85	6.54	1.79	8.55	3.52
Skewness	2.06	1.24	0.79	0.85	1.51	1.12	2.98	2.33
Kurtosis	17.05	13.85	5.91	6.76	11.65	9.58	27.45	32.21
Wiener entropy	0.43	0.06	0.88	0.09	0.65	0.12	0.76	0.19
Minimum fundamental frequency [kHz]	17.70	3.03	NA	NA	NA	NA	13.81	6.35
Time of minimum fundamental frequency [s]	0.11	0.06	NA	NA	NA	NA	0.01	0.02
Maximum fundamental frequency [kHz]	27.69	3.36	NA	NA	NA	NA	25.22	6.62
Time of maximum fundamental frequency [s]	0.01	0.02	NA	NA	NA	NA	0.01	0.01
Mean fundamental frequency [kHz]	21.47	3.21	NA	NA	NA	NA	19.06	5.39
Standard deviation of the fundamental frequency [kHz]	2.32	0.71	NA	NA	NA	NA	3.96	2.76
Mean slope [kHz/s]	273.28	83.45	NA	NA	NA	NA	1182.24	722.08
Frequency of first formant [kHz]	NA	NA	17.03	3.09	19.18	2.90	NA	NA
Bandwidth of first formant [kHz]	NA	NA	5.63	4.85	2.80	5.62	NA	NA
Frequency of second formant [kHz]	NA	NA	26.15	2.88	27.28	4.15	NA	NA
Bandwidth of second formant [kHz]	NA	NA	5.18	5.36	8.39	9.13	NA	NA
Frequency of third formant [kHz]	NA	NA	37.32	3.54	39.58	4.36	NA	NA
Bandwidth of third formant [kHz]	NA	NA	7.55	9.23	4.84	8.34	NA	NA

PUPS								
	**Screams** (N_{calls} = 53)		**Screeches** (N_{calls} = 7)		**Screech-Screams** (N_{calls} = 15)		**Chirps** (N_{calls} = 377)	
	**Mean**	**SD**	**Mean**	**SD**	**Mean**	**SD**	**Mean**	**SD**
Duration [s]	0.23	0.07	0.09	0.04	0.17	0.09	0.04	0.02
Time of peak amplitude [s]	0.10	0.07	0.04	0.03	0.03	0.02	0.02	0.01
Voiced percentage [%]	99.37	0.79	0.00	0.00	57.42	30.14	96.96	7.27
Harmonics-to-noise ratio	276.39	352.31	10.91	11.18	237.96	422.96	16.18	12.12
Centre of gravity [kHz]	31.82	4.30	22.15	2.74	28.45	4.56	19.86	4.19
Standard deviation [kHz]	6.21	2.18	7.64	1.95	6.30	2.55	8.14	2.01
Skewness	0.89	2.17	1.76	1.32	1.05	2.11	3.02	1.60
Kurtosis	29.63	31.16	9.57	5.25	20.00	15.70	22.30	19.27
Wiener entropy	0.39	0.05	0.71	0.06	0.55	0.12	0.76	0.11
Minimum fundamental frequency [kHz]	23.72	7.71	NA	NA	NA	NA	11.21	3.45
Time of minimum fundamental frequency [s]	0.20	0.08	NA	NA	NA	NA	0.01	0.02
Maximum fundamental frequency [kHz]	38.32	4.49	NA	NA	NA	NA	28.74	5.20
Time of maximum fundamental frequency [s]	0.03	0.05	NA	NA	NA	NA	0.02	0.01
Mean fundamental frequency [kHz]	31.34	4.85	NA	NA	NA	NA	20.06	4.15
Standard deviation of the fundamental frequency [kHz]	2.83	1.22	NA	NA	NA	NA	5.90	2.04
Mean slope [kHz/s]	595.64	216.46	NA	NA	NA	NA	958.10	316.76
Frequency of first formant [kHz]	NA	NA	17.55	2.05	18.00	5.07	NA	NA
Bandwidth of first formant [kHz]	NA	NA	4.16	3.08	2.90	3.37	NA	NA
Frequency of second formant [kHz]	NA	NA	27.68	4.96	28.48	3.49	NA	NA
Bandwidth of second formant [kHz]	NA	NA	7.24	7.38	4.32	5.13	NA	NA
Frequency of third formant [kHz]	NA	NA	38.59	6.56	39.44	4.92	NA	NA
Bandwidth of third formant [kHz]	NA	NA	9.44	9.55	5.26	4.61	NA	NA

Tab. S10 Results of the LME models testing which predictors (sex composition of the dyads. housing type and familiarity) had significant effects on the call rates. Bold p-values represent a significant effect $p < 0.05$. df = degrees of freedom.

Predictors	χ^2	df	p-value
**Screams**			
Sex composition of the dyad	0.93	2	0.627
Housing type	4.34	1	**0.037**
Familiarity	0.79	1	0.375
**Screeches**			
Sex composition of the dyad	0.31	2	0.858
Housing type	4.62	1	**0.032**
Familiarity	1.09	1	0.297
**Screech-screams**			
Sex composition of the dyad	0.80	2	0.669
Housing type	3.83	1	**0.050**
Familiarity	0.75	1	0.386
**Chirps**			
Sex composition of the dyad	6.95	2	**0.031**
Housing type	3.56	1	0.059
Familiarity	0.10	1	0.754

Tab. S11 Mean call rates by call types and influencing factors (sex composition of the dyads. housing type and familiarity).

	Screams [N_{calls}/min]	Screeches [N_{calls}/min]	Screech-screams [N_{calls}/min]	Chirps [N_{calls}/min]
**Sex composition of the dyads**				
Female-female	0.69 ± 2.24	0.48 ± 0.99	1.39 ± 4.17	0.81 ± 2.28
Male-female	0.47 ± 1.56	0.49 ± 0.79	1.09 ± 3.16	1.49 ± 3.11
Male-male	0.38 ± 1.21	0.39 ± 0.57	0.58 ± 1.37	0.16 ± 0.45
**Housing type**				
Pairs	1.01 ± 2.28	0.69 ± 1.00	1.91 ± 4.25	0.38 ± 0.66
Same sex groups	0.02 ± 0.06	0.24 ± 0.39	0.20 ± 0.42	1.55 ± 3.37
**Familiarity**				
Known	0.21 ± 0.57	0.29 ± 0.83	0.47 ± 1.17	0.93 ± 2.06
Unknown	0.64 ± 1.96	0.54 ± 0.76	1.30 ± 3.63	1.00 ± 2.71

Tab. S12 Settings for the analysis with Praat. In brackets it is noted for which script the settings were used. If for a setting there is no description in brackets, then these settings were used for all scripts. [1]the formant settings were calculated based on a vocal tract length of 1.2 cm. measured in an Etruscan shrew carcass according to Scheumann et al. (2012)[88]. Since pups produced non-tonal calls mainly in the second age class, where they were similar in size to adults, the same formant settings for both age classes were used.

**Spectrogram settings**	View range [Hz]	0 – 100.000
	Window length [s]	0.001
	Dynamic range [dB]	80
**Pitch settings**	Frequency range [Hz]	3.000 – 60.000
**(tonal calls)**	Time step [s]	0.001
**Formant settings[1] –**	Maximum formant [Hz]	80.000
**5 formants**		
**(non-tonal calls)**	Number of formants	5
	Window length [s]	0.003
	Dynamic range [dB]	30.0
	Dot size [mm]	1.0
**Voice report**	Period floor [s]	0.03
	Period ceiling [s]	0.45
	Maximum period factor	1.3
	Maximum amplitude factor	1.6
**Harmonicity – cross-correlation**	Time step [s]	0.001
**Analysis**	Minimum pitch [Hz]	5.000
	Silence threshold	0.1
	Number of periods per window	1
**Wiener entropy**	Frame duration [s]	0.001
	Time step [s]	0.004
	Frequency range [Hz]	5.000 – 50.000
